# Isolation of a planar π-aromatic Bi_5_^−^ ring in a cobalt-based inverse-sandwich-type complex

**DOI:** 10.1038/s41557-024-01713-8

**Published:** 2025-01-20

**Authors:** Julia Rienmüller, Benjamin Peerless, Sagar Paul, Florian Bruder, Wolfgang Wernsdorfer, Florian Weigend, Stefanie Dehnen

**Affiliations:** 1 Institute of Nanotechnology, Karlsruhe Institute of Technology, Karlsruhe, Germany; 2 Physikalisches Institut, Karlsruhe Institute of Technology, Karlsruhe, Germany; 3 Fachbereich Chemie, Philipps-Universität Marburg, Marburg, Germany; 4 Institute of Quantum Materials and Technology, Karlsruhe Institute of Technology, Karlsruhe, Germany

**Keywords:** Chemical bonding, Ligands, Synthetic chemistry methodology, Density functional theory

## Abstract

Monocyclic π-aromatic compounds are ubiquitous throughout almost all fields of natural sciences—as synthons in industrial processes, as ligands of metal complexes for catalysis or sensing and as bioactive molecules. Planar organocycles stand out through their specific way of overcoming electron deficiency by a non-localizable set of (4*n* + 2)π electrons. By contrast, all-metal aromatic monocycles are still rare, as metal atoms prefer to form clusters with multiply bonded atoms instead. This limits the knowledge and potential of corresponding compounds in chemical syntheses or for innovative materials. Here we report the successful generation of Bi_5_^−^, the heaviest analogue of (C_5_H_5_)^−^. Its use as a ligand in [{IMesCo}_2_(µ,η^5^:η^5^-Bi_5_)] (1) was realized by reacting (TlBi_3_)^2−^ with [(IMes)_2_CoCl] (where IMes is bis(1,3-(2,4,6-trimethylphenyl))imidazol-2-ylidene) in *ortho-*difluorobenzene. Compound 1 is mixed-valence Co^0^/Co^I^ as verified by µ-SQUID measurements and density functional theory, and embeds the planar Bi_5_^−^ cycle in an inverse-sandwich-type manner. Capturing Bi_5_^−^ represents a landmark in the chemistry of all-metal aromatic molecules and defines a new era for aromatic compounds.

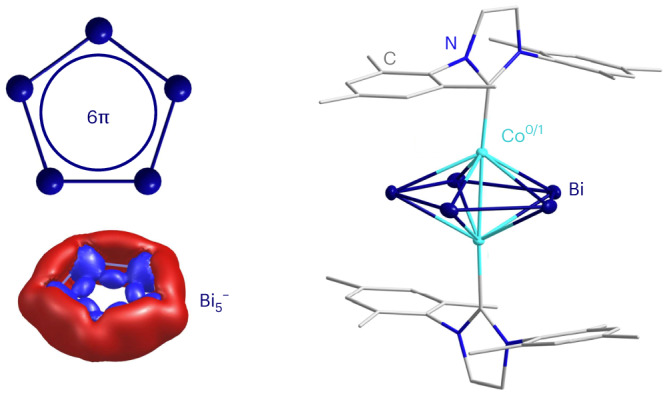

## Abstract (web summary)

All-metal aromatic monocycles are still rare, in contrast to their ubiquitous organic counterparts, because metal atoms tend to form clusters with multiply bonded atoms instead. Now a planar aromatic Bi_5_^−^ ring has been synthesized as part of a mixed-valence Co^0^/Co^I^ inverse-sandwich-type complex.

## Main

Ligands composed of π-aromatic five-membered rings are ubiquitous throughout transition metal chemistry, with the anionic (C_5_H_5_)^−^ (cyclopentadienide) ligand occupying the top spot in usage since the elucidation of the structure of ferrocene^1,2^. From sandwich, half-sandwich, inverse-sandwich and triple-decker complexes with the ring acting as η^5^-, η^1^- and η^3^-type ligands, this ligand class spans almost all fields of the chemical sciences—in catalysis, redox and photochemical sensors, and bioactive compounds, to name a few^3–9^. Exchanging a CR group (R = H or organic substituent) from the general formula (C_5_R_5_)^−^ with a valence isoelectronic group-15 element (Pn) affords (C_5−*x*_R_5−*x*_Pn_*x*_)^−^. The fully exchanged species, Pn_5_^−^ (*x* = 5), represents a purely inorganic ligand; so far, the synthetic realization of such ligands has been possible for all pnictogens^10–13^ except the bismuth congener, which has therefore remained a major challenge and unsolved task in synthetic chemistry. A {Bi_5_} species denoted as Bi_5_^3−^ had been detected as early as in 1931 during the potentiometric titration of Na in liquid NH_3_ with a BiI_3_ solution by the name patron of polymetallide ‘Zintl’ anions, Eduard Zintl, but it could not be isolated^14^. Notably, the direct group-14 analogue—the Zintl anion Pb_5_^6−^—was reported in a complex with two {Mo(CO)_3_}^+^ units^15^, but the electronic situation could not be clarified in full and allowed several different interpretations.

The enhanced metallic character of bismuth often hinders viable starting materials for Bi_5_^−^, which is not a problem for the lighter elements of the group. The pentazolate anion, N_5_^−^, can be prepared from C–N bond cleavage of arylpentazoles using *meta*-chloroperoxybenzoic acid and transition metal complexes as stabilizers^13^, the pentaphosphacyclopentadienide and pentaarsacyclopentadienide anions, P_5_^−^ and As_5_^−^, can be prepared from the reduction of P_4_ and As_4_ tetrahedra, respectively^16,17^, and the pentastibacyclopentadienide analogue, Sb_5_^−^, has been prepared starting from *t*Bu_4_Sb_4_ (ref. ^12^).

With the recent emphasis on the development of bismuth chemistry over the past couple of years^18–20^, the number of accessible and versatile sources for all-bismuth molecules as ligands has been increasing (namely, polybismuthides)^21–24^. There are two common methods for transferring such anionic bismuth clusters into solution: either by extraction of a binary or ternary intermetallic solid with composition K_*x*_Bi_*z*_ or K_*x*_T_*y*_Bi_*z*_ (T = Tr = group-13 or T = Tt = group-14 element, with *x*/*y*/*z* being K/Bi, 5/4; K/Ga/Bi, 5/2/4; K/In/Bi, 5/2/4; K/Tl/Bi, 2/1/3; K/Ge/Bi, 2/1/1; K/Sn/Bi, 1/1/1; K/Pb/Bi, 1/1/1) in the presence of 4,7,13,16,21,24-hexaoxa-1,10-diazabicyclo[8.8.8]hexacosane (crypt-222) and ethane-1,2-diamine (en), or by dissolution of salts of bismuth-containing anionic tetrahedra, [K(crypt-222)]_2_(GaBi_3_)·en, [K(crypt-222)]_2_(InBi_3_)·en, [K(crypt-222)]_2_(TlBi_3_)·0.5en, [K(crypt-222)]_2_(Sn_2_Bi_2_)·en and [K(crypt-222)]_2_(Pb_2_Bi_2_)·en, in polar solvents^25–28^. Each method has yielded a plethora of homoatomic and, when combined with *d*-block or *f*-block metal complexes, heteroatomic polybismuthides, typically as anionic cluster molecules (for related investigations towards polycationic polybismuth molecules that are not addressed herein, see refs. ^17,29–34^). The value of *n* in {Bi_*n*_} moieties that make up these compounds ranges from as low as 2, Bi_2_^2−^ (ref. ^35^), and reaching the current record, which stands at 18 bismuth atoms in [{Ru(cod)}_4_Bi_18_]^4−^ (ref. ^36^). Yet, among species containing fewer than ten Bi atoms^21,22,24,28,37–44^, {Bi_5_} remains elusive in isolable compounds, which is remarkable, as the five-ring motif is predominant in the structural chemistry of pnictogens, and as Bi_5_^−^ was also detected in photoelectron spectra upon generation in a pulsed-arc cluster ion source^45^ and as a fragment of high abundance in mass spectra of polybismuthide solutions^36^. Now, as the knowledge in the formation pathways of polybismuthides expands^36,38^, specific targeted synthesis is also starting to become possible.

Extraction solutions of intermetallic solids comprising bismuth or solutions of binary *p*-block metal/Bi anions provide suitable reaction environments for (1) the removal of ligands from an organometallic reactant and (2) the synthesis of polybismuthide frameworks. Group-14/Bi anions often retain both metals in the subsequent cluster formed, whereas group-13/Bi anions tend to release the group-13 element and provide small anionic {Bi_*n*_} fragments that often aggregate further to larger {Bi_*n*_} moieties. Based on this recent experience, we suspected that Bi_2_^2−^ or (TrBi_3_)^2−^ anions represent the best starting material for the generation of the long-sought Bi_5_^−^ moiety.

Here, we report the success of this synthetic concept by filling in this gap and completing the series of pentapnictacyclopentadienide ligands with the isolation of an inverse-sandwich complex-type cluster containing this unique planar {Bi_5_} ring. First, we identified the existence of the Bi_5_^−^ anion in solution by mass spectrometry after titration of [K(crypt-222)]_2_Bi_2_ with BiI_3_ in en^14,46,47^. Density functional theory (DFT) calculations confirmed its monocyclic structure and π-aromatic nature. Subsequently, we obtained the {Bi_5_} moiety in an isolable compound from the reaction between [K(crypt-222)]_2_[TlBi_3_]∙0.5en and [(IMes)_2_CoCl] (where IMes is bis(1,3-(2,4,6-trimethylphenyl))imidazol-2-ylidene). The replacement of en with *ortho*-difluorobenzene (*o*-DFB) as an uncommon solvent in Zintl chemistry enabled the crystallization of [{IMesCo}_2_(µ,η^5^:η^5^-Bi_5_)] (1), representing the first isolable complex of the Bi_5_^−^ ligand. Different from all reported reaction products of Zintl anions with *d*-block or *f*-block metal complexes, compound 1 is a neutral compound. In addition, it is a rare case of a mixed-valent Co^0^/Co^I^ complex, which was corroborated by a combination of highly sophisticated micron size superconducting quantum interference device (µ-SQUID) and DFT studies. The compound serves to explain the stability of such open-shell complexes in comparison with a hypothetical, anionic closed-shell analogue.

## Results and discussion

### Solution studies of Bi_5_^−^ and computations on its aromaticity

**Fig. 1:**
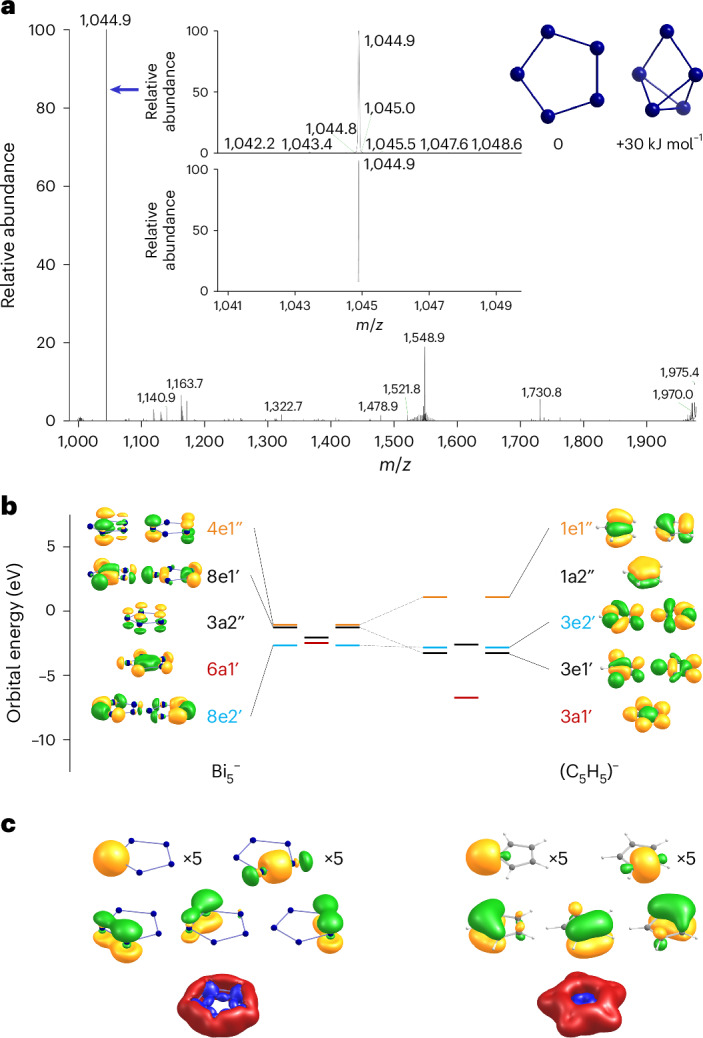
Mass spectrometric proof of the Bi_5_^−^ anion in solution as well as its molecular and electronic structure including π-aromatic features according to quantum chemical studies in comparison with (C_5_H_5_)^−^. **a**, ESI mass spectrum in negative ion mode of the reaction solution prepared from [K(crypt-222)]_2_Bi_2_ and BiI_3_ (2:1) in en, with the molecular peak of Bi_5_^−^ at *m*/*z* = 1,044.9 being the clearly predominant signal; the inset spectrum zooms into the high resolution of the mass peak as measured (top) and calculated (bottom). The molecules shown top right are the two most energetically favourable isomers of Bi_5_^−^, a planar ring (*D*_5*h*_), and a capped butterfly-type isomer (*C*_2*v*_, +30 kJ mol^−1^), according to DFT calculations. **b**, Energies and amplitudes of *p*-orbital-based valence MOs of Bi_5_^−^ (left) and (C_5_H_5_)^−^ (right), with different superatomic MO types (albeit of a two-dimensional molecule) specified by different colours of bars and Mulliken symbols: *s*-type (red), *p*-type (black), *d*-type (orange) and *f*-type (blue); contours are drawn at ±0.04 a.u.; full-valence MO diagrams are shown in Supplementary Fig. 1. **c**, Top and centre: amplitudes of LMOs of Bi_5_^−^ (left) and (C_5_H_5_)^−^ (right); contours are drawn at ±0.05 a.u.; in the upper row, only one representative of each of the five equivalent LMOs is shown. Bottom: contour plot of the absolute value of the magnetically induced current density, drawn at 0.025 a.u.; contours for paratropic currents are drawn in blue, and those for diatropic currents are drawn in red.

From as early as the pioneering work of Zintl, the existence of a pentaatomic polybismuthide anion was postulated by potentiometric titration experiments involving BiI_3_. Therefore, [K(crypt)]_2_Bi_2_ was treated with BiI_3_ to emulate such an experiment in a more synthetic manner. A 2:1 mixture of the two compounds, respectively, was dissolved in en. Electrospray ionization mass spectrometry (ESI-MS) of the dark-green–blue supernatant showed that the most dominant peak in negative ion detection mode appeared at 1,044.9 *m*/*z*, fitting perfectly with the assignment of Bi_5_^−^. The overview mass spectrum and the high-resolution molecular peak are shown in Fig. 1a.

As mass spectrometry alone does not allow to distinguish between a ring-type and a cluster-type structure, quantum chemical calculations^48^ were done for the Bi_5_^−^ anion, also in comparison with the lighter homologues of the Pn_5_^−^ series (Pn = P, As, or Sb). The Perdew-Burke-Ernzerhof (PBE) functional^49^ was used with dhf-TZVP basis sets^50^ together with corresponding effective core potentials^51^ for Bi. By a genetic algorithm procedure^52^, a planar five-membered ring was identified as the global minimum of the energy surface, and the second most stable one is a capped butterfly structure, which is higher in energy by 30 kJ mol^−1^ (Fig. 1a, inset). Standard DFT functionals as well as Hartree–Fock and second-order Møller–Plesset perturbation theory (MP2) reproduce this value typically within ±10 kJ mol^−1^ (Supplementary Table 1). Inclusion of spin–orbit coupling (SOC)^53^ increases the preference for the ring to 66 kJ mol^−1^; this substantial influence of SOC on the energy surface is typical for Bi clusters^54^. The equilateral {Bi_5_} pentagon exhibits Bi–Bi distances of 2.88 Å (2.92 Å considering SOC; the elongating effect of the SOC for Bi was discussed by van Lenthe et al. many years ago^55^). We further note that the energetic preference for the ring is more pronounced for the lighter homologues; for Sb, As and P, it amounts to 46, 110 and 141 kJ mol^−1^ (calculated without SOC), respectively. The highest occupied molecular orbitals (MOs) of Bi_5_^‒^ are formed by the 6*p* orbitals. They are analogous to those of (C_5_H_5_)^‒^, yet with different energy distances and also another order in energy of *s*-type, *p*-type, *d*-type and *f*-type two-dimensional superatomic MOs (Fig. 1b and Supplementary Fig. 1). They can be transformed by a Pipek–Mezey MO localization procedure^56^ to five Bi–Bi two-centre single bonds and three π-type bonds, and a further five lone pairs/C–H bonds (Fig. 1c, top and centre). The presence of a delocalized π-system (six π-electrons at five atoms) implies the occurrence of magnetically induced ring currents, which have been calculated from the magnetic response density with gauge-including magnetically induced current (GIMIC)^57^. They are very similar for Bi_5_^−^ and (C_5_H_5_)^−^, with values of 14.4 nA T^−1^ and 12.8 nA T^−1^—reflecting aromaticity for both systems, as visualized in Fig. 1c (bottom). This is similar to Sb_5_^−^, As_5_^−^ and P_5_^−^ with 17.6, 18.6 and 19.6 nA T^−1^, respectively. We therefore attribute the preference of the ring-type structure of Pn_5_^−^ over the capped butterfly-type isomer at least in part to the stabilization by the 6π-Hückel aromaticity. The ring current is much smaller for the threefold positively charged 2π-species, that is, if the degenerate highest occupied molecular orbital (HOMO, Fig. 1b) is empty. It amounts to 5.9 nA T^−1^ for (C_5_H_5_)^3+^ and to 2.8, 6.2, 8.1 and 10.0 nA T^−1^ for Bi_5_^3+^, Sb_5_^3+^, As_5_^3+^ and P_5_^3+^, respectively, pointing to an increasing relevance for aromaticity of these two degenerate π-orbitals from P to Bi. The 6*s* orbitals of Bi_5_^−^ are much lower in energy (by ~7 eV) and are not relevant for the bonding. For completeness, we also list the nucleus-independent chemical shifts at the ring centres of Bi_5_^−^, Sb_5_^−^, As_5_^−^, P_5_^−^ and (C_5_H_5_)^−^, which amount to −9.0, −11.5, −15.2, −15.4 and −13.5 ppm, respectively, which also reflect the similarities in the aromatic behaviour of these compounds.

Attempts to crystallize a compound from such solutions and trapping experiments with transition metal complexes have been challenging and remain ongoing, as appropriate conditions for these subsequent steps are yet to be found. However, based on the knowledge that this heaviest analogue of (C_5_H_5_)^−^ can be prepared and exists in solution, we considered alternative experimental approaches to form and capture Bi_5_^−^ by variation of (1) the source of bismuth and (2) the solvent for in situ reactions with transition metal compounds.

### Synthesis and structure of [{IMesCo}_2_(µ,η^5^:η^5^-Bi_5_)] (1)

We succeeded in trapping the Bi_5_^−^ anion by performing reactions of (TrBi_3_)^2−^ anions (Tr = Ga, In or Tl), which were reported to occasionally release the Tr atom under formation of Bi-richer substructures in situ in the presence of transition metal complexes. Typically, en is the solvent of choice, but *o*-DFB has shown great promise as an alternative for this chemistry. The stability of (TrBi_3_)^2−^ in *o*-DFB was found to be limited following the trend Ga < In < Tl as least stable to most stable, on the basis of the time taken for the intensely coloured solutions to become colourless, and concurrent precipitation of black powders. As an outcome of these studies, the stability of (TlBi_3_)^2−^ in *o*-DFB was found to be sufficiently high enough for reactions with transition metal complexes, but at the same time, the tendency of ultimately releasing Tl is high enough for successfully acting as a source of Bi_5_^−^, although this is seemingly not possible for the two others, as either the former or the latter is less favourable.

**Fig. 2:**
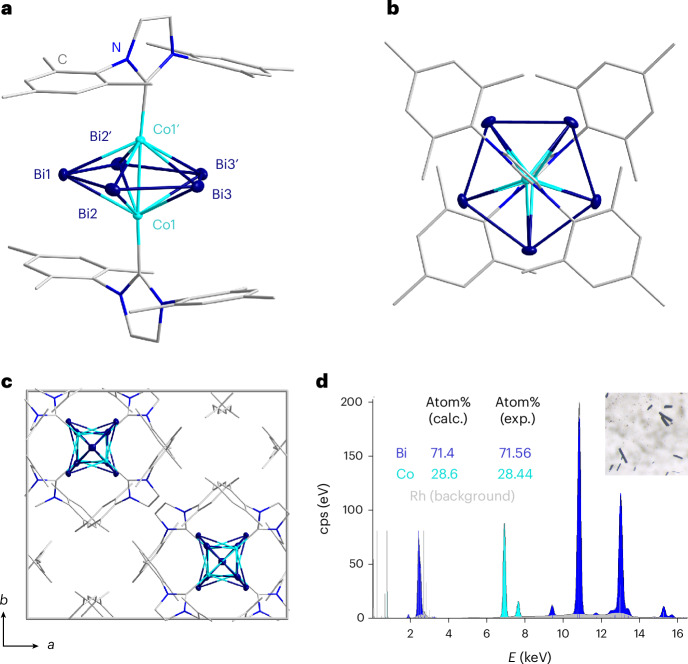
Crystal structure and metal-atom composition of [{IMesCo}_2_(µ,η^5^:η^5^-Bi_5_)] (1). **a**, The molecular structure of 1 from a side view onto the Co···{Bi_5_}···Co pentagonal bipyramid indicating the colour code used for the atom types. H atoms are not shown for clarity. Selected atomic parameters are given in the text. **b**, The molecular structure of 1 from a top view onto the {Bi_5_} moiety. **c**, The unit cell plot of 1 viewed down the crystallographic *c* axis. The channels running along the *c* axis at ¼, ¼, *z* and ¾, ¾, *z* do not contain any electron density beside the H atoms at the IMes ligand. **d**, A µ-XRF spectrum recorded on single crystals of 1 (see light-microscopic image in the inset) with the results of the deconvolution algorithm given in counts per second (cps) versus energy (*E*), indicating the perfect match of the calculated (calc.) and observed (exp.) compositions. The colour code refers to the colours used in the crystal-structure figures: Bi (blue) and Co (turquoise).

The reaction between [(IMes)_2_CoCl] and [K(crypt-222)]_2_TlBi_3_∙0.5en in *o*-DFB resulted in a brown solution immediately with a small amount of black precipitate. In situ ESI-MS measurements (Supplementary Figs. 2–5) showed two relevant signals at 1,044.9 *m*/*z* and 1,771.1 *m*/*z* for Bi_5_^−^ and a compound that could be assigned to the chemical formula of the anionic species [IMes_2_Co_2_Bi_5_]^−^, respectively, after 2 h. Subsequent work-up afforded an appreciable quantity of dark-brown plate-like crystals of excellent crystal quality over a period of 1 month (Supplementary Fig. 6), identified as the neutral complex [{IMesCo}_2_(µ,η^5^:η^5^-Bi_5_)] (1) by means of single-crystal X-ray diffraction and microanalysis. The result of the X-ray structure analysis of 1 is given in Fig. 2 and Supplementary Table 2. The crystallographic symmetry of the molecule is *C*_2_, with the axis running through Bi1 and the centre of the Bi3–Bi3′ bond. Supplementary images also displaying H atoms and the full labelling scheme are provided in Supplementary Figs. 7 and 8.

The [{IMesCo}_2_(µ,η^5^:η^5^-Bi_5_)] molecule has an inverse-sandwich-type structure with an almost planar η^5^-Bi_5_ ring situated between two Co atoms that sit above and below the centre of the ring (Fig. 2a,b). The Bi–Bi bond lengths are remarkably similar (2.9035(3)–2.9097(4) Å) and lie between those of a Bi–Bi double bond and Bi–Bi single bond (cf. 2.8377(7) Å in Bi_2_^2−^ and 2.990(2) Å in Ph_4_Bi_2_)^35,58^, consistent with the idea of a delocalized structure akin to the valence isoelectronic (C_5_H_5_)^−^ and similar to the other Pn_5_^−^ congeners, as outlined above. The Co–{Bi_5_}_centroid_–Co′ angle is nearly linear, 179.27(3)°. The two IMes ligands sit in a staggered conformation with respect to one another, most likely to minimize steric repulsion in the overall crystal lattice, and are only slightly bent away from the Co–Co′ axis with a Co–Co–C angle of 175.97(9)°. The distance between the two Co atoms, 2.5157(8) Å, is below the sum of the atoms’ covalent radii (2 × 1.26 = 2.56 Å)^59^, pointing to notable bonding interactions (see below).

Different from other heterometallic compounds obtained from reactions of Zintl anions, the crystal structure refinement clearly only gives the complex molecule, which therefore needs to be neutral, not anionic. Channels that run through the crystal along the crystallographic *c* axis at ¼, ¼, *z* and ¾, ¾, *z* were found to contain no electron density beside that of the H atoms at the IMes ligands (Fig. 2c). As naked H^+^ ions can hardly be reasonably stabilized within such channels that are formed exclusively by rather non-polar organic groups, and as micro-X-ray fluorescence (µ-XRF) spectroscopy (Fig. 2d and Supplementary Table 3) indicates that there is no other component that could form or act as a cation, for example, none of the otherwise typical [K(crypt-222)]^+^ cations, we can exclude that compound 1 contains any further components beside the [{IMesCo}_2_(µ,η^5^:η^5^-Bi_5_)] molecule.

### Electronic structure and magnetism of 1

The neutral nature of complex 1 is highly uncommon for compounds composed of purely inorganic Zintl anions and metal complexes^18,34,60–66^, and it leads to an electronic situation that raises interesting questions. On the basis of pseudo-element considerations, the {Bi_5_} ligand should have a 1− charge and, therefore, the distinct lack of a counterion would afford the two Co atoms an assignment of the 0 and +I oxidation states as a mixed-valence complex and, subsequently, an open-shell compound. Besides this plausibility-based conclusion, the very uncommon electronic features and the bonding within compound 1 were confirmed in multiple ways.

First, we refer to the known (mostly homoleptic) inverse-sandwich complexes that comprise homologues or analogues of the Bi_5_^−^ anion, namely P_5_^−^, As_5_^−^ and Sb_5_^−^, and (P_4_Tt)^2−^ (Tt = Sn, Pb). Remarkably, except for two cationic (and heteroleptic) species, in [(η^5^-C_5_H_5_)Fe(μ,η^5^:η^5^-P_5_)Fe(η^5^-C_5_Me_4_Et)][PF_6_] (ref. ^67^) and [(η^5^-C_5_H_5_)Fe(μ,η^5^:η^5^-As_5_)Fe(η^5^-C_5_Me_5_)][PF_6_] (ref. ^17^), all other compounds were reported to be neutral, too, namely, [{(C_5_R_4_R’)Cr}_2_(μ,η^5^:η^5^-P_5_)] (R/R′ = H/H, H/Me or Me/Me) (ref. ^10^), [{IMesNi}_2_(μ,η^5^:η^5^-P_5_)], [{IPrNi}_2_(μ,η^5^:η^5^-P_5_)] (ref. ^68^), [{(C_5_H_5_)Mo}_2_(μ,η^5^:η^5^-As_5_)] (ref. ^11^), [{(η^5^-C_5_R_4_R’)Cr}_2_(μ,η^5^:η^5^-As_5_)] (R/R′ = Me/Me or Me/Et) (ref. ^69^), [(C_5_Me_5_)M(μ-η^5^:η^5^-E_5_)M′(CO)_3_] (M/E/M′ = Fe/P/Mo, Fe/As/Cr or Fe/As/Mo) (ref. ^70^), [{(η^5^-1,2,4-*t*Bu_3_C_5_H_2_)Mo}_2_(μ,η^5^:η^5^-Sb_5_)], [{(η^5^-1,2,4-*t*Bu_3_C_5_H_2_)Mo}(μ,η^5^:η^5^-Sb_5_){(η^5^-1,4-*t*Bu_2_-2-MeC_5_H_2_)Mo}] (ref. ^12^) and [{(η^4^-*t*Bu_2_C_2_P_2_)Co}_2_(μ,η^5^:η^5^-P_4_Tt)] (ref. ^40^). The bonding situation of the first {As_5_}-based species was hypothesized to be based on an As_5_^4−^ ring and two [(C_5_H_5_)Mo^III^]^2+^ moieties—which later turned out to be incorrect and which also disagrees with our own calculations on this species. Some of the other compounds, namely, [{(C_5_Me_5_)Cr}_2_(μ,η^5^:η^5^-P_5_)], [{IPrNi}_2_(μ,η^5^:η^5^-P_5_)], [{(C_5_H_5_)Mo}_2_(μ,η^5^:η^5^-As_5_)], [{(η^5^-1,2,4-*t*Bu_3_C_5_H_2_)Mo}_2_(μ,η^5^:η^5^-Sb_5_)] and [{(η^5^-1,2,4-*t*Bu_3_C_5_H_2_)Mo}(μ,η^5^:η^5^-Sb_5_){(η^5^-1,4-*t*Bu_2_-2-MeC_5_H_2_)Mo}], were explicitly reported to be paramagnetic and discussed as comprising analogues of the (C_5_H_5_)^−^ ligand. This is a strong indication that the situation in compound 1, opening the series containing the heaviest Bi-based cousin of (C_5_H_5_)^−^, is also mixed valence.

**Fig. 3:**
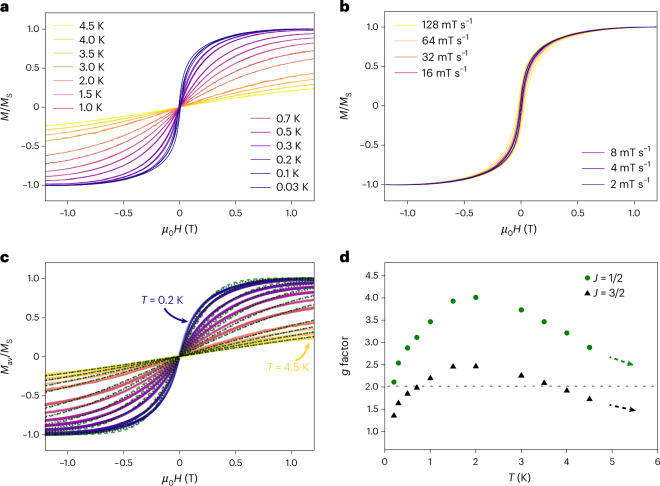
Results of the µ-SQUID measurements on single crystals of [{IMesCo}_2_Bi_5_] (1). **a**,**b**, Normalized *M*(*H*) loops obtained at different bath temperatures with fixed field sweep rate of 0.016 T s^−1^ (**a**) and fixed bath temperature *T* = 30 mK with different field sweep rates (**b**). **c**, Average and symmetric *M*_av_/*M*_S_(*H*) curves (solid lines) obtained from forward and backward field cycles fitted to Brillouin functions. The dashed green and dotted black lines show the fittings with *J* = 1/2 and 3/2, respectively. To fit each curve, the spin temperature is assumed to be equal to the corresponding bath temperature *T*. **d**, The *g* factors obtained from the fittings in **c** are shown for the two cases *J* = 1/2 and 3/2. The dotted line indicates the *g* factor value calculated by DFT studies (see below).

Second, we carried out µ-SQUID measurements (Methods) on single crystals at sub-Kelvin temperatures to corroborate the paramagnetic nature of [{IMesCo}_2_Bi_5_] (1). The temperature and field sweep-rate-dependent *M*(*H*) curves in Fig. 3a,b show clear saturation fields and a sufficiently large µ-SQUID signal to indicate the spin of the magnetic ions. The sweep-rate-dependent *M*(*H*) curves measured at 30 mK exhibit hysteresis for higher sweep rates (Fig. 3b), suggesting mild intra- or intermolecular interaction. It is most likely dominated by an intramolecular Co–Co interaction. The shape of the *M*(*H*) curve, dominated by a sharp transition about zero field, indicates a ferromagnetic nature of the interaction since an antiferromagnetic interaction usually shows a flat *M*(*H*) about zero field between two sharp transitions^71^. A considerable sweep rate dependence of the *M*(*H*) indicates low tunnel splitting in the energy (Zeeman) diagram about zero field and thermally dominated relaxation. Moreover, the phonon bottleneck effect^72,73^ possibly contributes to the hysteresis for such a low-spin system with relaxation mediated by single phonon excitation processes.

For temperatures higher than 0.2 K, the hysteresis vanishes, yielding a paramagnetic nature (Fig. 3a). The paramagnetic experimental curves are used to obtain the symmetric and average *M*_av_(*H*) curves by averaging over the forward and backward field cycles of *M*(*H*), as shown in Fig. 3c. These are fitted to Brillouin functions, equation (1)^74^, as follows:

1 $$M/{M}_{{\mathrm{S}}}={B}_{J}(x)=\frac{2J+1}{2J}\,\coth \left(\frac{2J+1}{2J}x\right)-\frac{1}{2J}\,\coth \left(\frac{1}{2J}x\right),$$

with *x* = *Jgµ*_B_*µ*_0_*H/*(*k*_B_*T*_spin_). Here, a ground state *J* value is assumed to obtain the *g* factor, while the spin temperature *T*_spin_ is assumed to be equal to the corresponding bath temperature *T* for *T* ≥ 0.2 K. A good fitting quality with reduced *R*^2^ > 0.996 (for *J* = 1/2) and >0.997 (for *J* = 3/2) confirms the paramagnetic nature. Only the fractional *J* values are considered when expecting an odd number of unpaired electrons in the mixed-valence compound.

Figure 3d shows the *g* factors obtained from the fittings, for these two cases, *J* = 1/2 and 3/2. Although the fitting quality is slightly better for the case *J* = 3/2, Fig. 3d indicates *J* = 1/2 as the most plausible scenario for the following reasons. First, in most molecular magnets, the quantum tunnelling of magnetization at the zero-field anti-level crossing between the magnetic states notably influences the shape of the low-temperature *M*(*H*) curves. Second, the assumption *T*_spin_ = *T* is not accurate as it depends on thermalization of the crystal to the substrate, and the phonon bottleneck effect^72,73^ can cause large differences between *T*_spin_ and *T*. Third, a small contribution of a Raman relaxation process can also mildly affect the experimental *M*(*H*) curve in transition-metal-based systems. These effects, not considered within the Brillouin function, lead to substantial mismatch between the experimental data and the fittings, and large deviations of fitted *g* factors from the actual value. With increasing *T*, an abrupt increase, followed by a slow decrease of fitted *g* values, indicates that multiple relaxation mechanisms are at play. A saturation to the correct *g* value is expected towards higher *T* (Fig. 3d, arrows), since the mentioned effects contribute negligibly at elevated temperatures. The usual paramagnetic scenario, described solely by the Brillouin function, is regained at higher temperatures until the excited spin states beyond several thousand Kelvin (see below) are accessible. Finally, the fitted *g* factor for *J* = 1/2 tends to saturate (Fig. 3d, green arrow) towards the value expected from quantum chemical calculations (*g* ≈ 2.03; Table 1). This rules out the assumption of *J* = 3/2 and higher spins in the Brillouin function, as they lead to non-reasonable saturation values of the fitted *g* factor.

**Table 1:** Geometric and electronic structure data of [{IMesCo}_2_Bi_5_] (1) and hypothetical anion [{IMesCo}_2_Bi_5_]^−^ obtained with the functional PBE

	Co–Co	Co–Bi	Bi–Bi	C–Co–Co	*E*	*S*^2^ (*S*^2^–*S*[*S* + 1])	*g*	Gap	*N*_ue_ (Co/Co/Bi_5_)
**X-ray**	2.52	2.75–2.79	2.90–2.91	176	–	–		–	–
**M**^**−**^	2.53	2.72–2.83	2.91–2.93	176		0		1.235	0.0/0.0/0.0
^**2**^**A**	2.56	2.70–2.88	2.90–2.96	170	0	0.811 (0.061)	2.03	0.254	0.49/0.49/0.03
^**2**^**B**	2.57	2.72–2.85	2.89–2.92	179	0	0.764 (0.014)	2.03	0.169	0.44/0.44/0.19
^**2**^**BS**	2.53	2.67–2.86	2.90–2.94	178/169	–3	0.830 (0.083)	2.05	0.331	1.20/–0.19/0.04
^**4**^**A**	2.65	2.76–2.95	2.93–2.94	167	+46	3.887 (0.137)	1.93	0.057	1.51/1.51/0.05

Column labelling: Co–Co, Co–Bi and Bi–Bi are the (ranges of) distances (in Å) between the corresponding atoms, C–Co–Co labels the bend of the Co–Co axis and the Co–C axes at the two Co atoms (in °). *E* is the energy relative to the ^2^A state (in kJ mol^−1^); in the following column, the *S*^2^ values and their deviations from the value for the pure doublet/quartet are given, *g* is the calculated *g* factor, Gap denotes the HOMO–LUMO gap (in eV) and *N*_ue_ is the number of unpaired electrons according to a Mulliken analysis^75^ at the Co atoms and at the {Bi_5_} ring. Row labelling: M^−^ denotes the anionic (diamagnetic) species. The subsequent lines denote neutral species: in ^2^A, the orbital 126a (HOMO within the irrep a) is singly occupied, in ^2^B, the orbital 123b (HOMO within irrep b) is singly occupied. ^2^BS is a broken symmetry state with overall one unpaired electron. In ^4^A the unpaired electrons reside in 126a, 123b and 124b (Fig. 5).

In summary, the presence of a notable µ-SQUID signal, the saturation observed in the measured *M*(*H*) and the fittings with the paramagnetic Brillouin functions are in full alignment with compound 1 being an open-shell compound with possibly *J* = *S* = 1/2, which rationalizes the formulation as a neutral Co^0^/Co^I^ mixed-valence complex.

Finally, we used quantum chemical calculations at the same level of theory outlined above, to clarify the electronic structure, in particular the role of the {Bi_5_} ring in the title compound. The computational treatment of [{IMesCo}_2_Bi_5_] (1) was a considerable challenge due to the sensitivity of the energetic level of the Co-*d* orbitals towards the functional used. For details on the solution of this problem, see Supplementary Information.

**Fig. 4:**
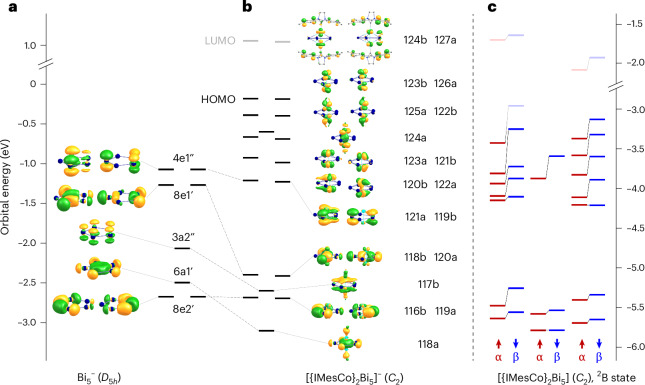
Electronic structure (frontier orbital scheme) of Bi_5_^−^, the hypothetical anion [{IMesCo}_2_Bi_5_]^−^ and the neutral complex [{IMesCo}_2_Bi_5_] (1), obtained from quantum chemical calculations. **a**, The eight highest occupied MOs of Bi_5_. **b**, The 17 highest occupied MOs (black bars) and two lowest unoccupied MOs (grey bars) of [{IMesCo}_2_Bi_5_]^−^. Contours are drawn at 0.04 a.u. The contributions of the IMes groups for the highest occupied MOs are very small; the atoms of these groups are thus omitted in the images, except for LUMO and LUMO+1, to which they contribute substantially. **c**, The 17 highest occupied alpha spin MOs (red bars) and 16 highest beta spin MOs (blue bars) as well as two lowest unoccupied alpha spin MOs (light-red bars) and three lowest unoccupied beta spin MOs (light-blue bars) of the open-shell complex [{IMesCo}_2_Bi_5_] in its ^2^B state (see also below). The grey lines between alpha and beta spin MOs illustrate the release of degeneracy as compared with the closed-shell MOs. Note that the energy levels of the two complexes naturally differ owing to the presence or absence of a negative charge.

As outlined above, [{IMesCo}_2_Bi_5_] (1) has an odd number of electrons. This is in contrast to its (hypothetical) anionic counterpart, [{IMesCo}_2_Bi_5_]^−^, which would be diamagnetic and was therefore used as starting point for the discussion, as 1 can be described by the removal of one electron from the anion. The frontier orbitals of the hypothetical anion are shown in relationship with those of Bi_5_^−^ (Fig. 4a, *D*_5*h*_ symmetry; same set of MOs as in Fig. 1b) in Fig. 4b, and compared with those of the neutral open-shell compound [{IMesCo}_2_Bi_5_] (1; ^2^B state) shown in Fig. 4c. *C*_2_ symmetry was assumed for the complexes, with the rotation axis running from left to right in the figure.

The highest nine occupied MOs of the anion are located mainly at the two Co atoms; the subsequent eight are clearly related to their counterparts in the bare {Bi_5_} ring, but most of them are substantially stabilized by binding admixtures of the Co(*d*) orbitals. HOMO and HOMO-1 are (near-)degenerate symmetric and anti-symmetric linear combinations of Co(*d*) orbitals that are in plane with the IMes rings, near-degenerate HOMO-2 and HOMO-3 are linear combinations of Co(d) orbitals perpendicular to the IMes rings, thus showing a slight interaction with the C(*p*) orbitals of the neighboured C atom of the ring; HOMO-4 (MO 124a) is the binding combination of Co(*d*) orbitals along the Co–Co axis. A fully localized description of the bonding situation is not possible, illustrating the inherently multicenter- and cluster-type nature of the {Co_2_Bi_5_} moiety. Localized MOs (LMOs) of a simplified model of the title compound, [(C_3_N_2_H_4_)Co}_2_Bi_5_], are shown in Supplementary Fig. 10. One finds five orbitals representing the σ-bonds between the Bi atoms and three further orbitals (six electrons) that represent the delocalized π-system that also shows contributions from the Co atoms. The calculated structure parameters for the anion, presented in Table 1, agree very well with the X-ray data for the neutral species, typically within 0.02 Å.

**Fig. 5:**
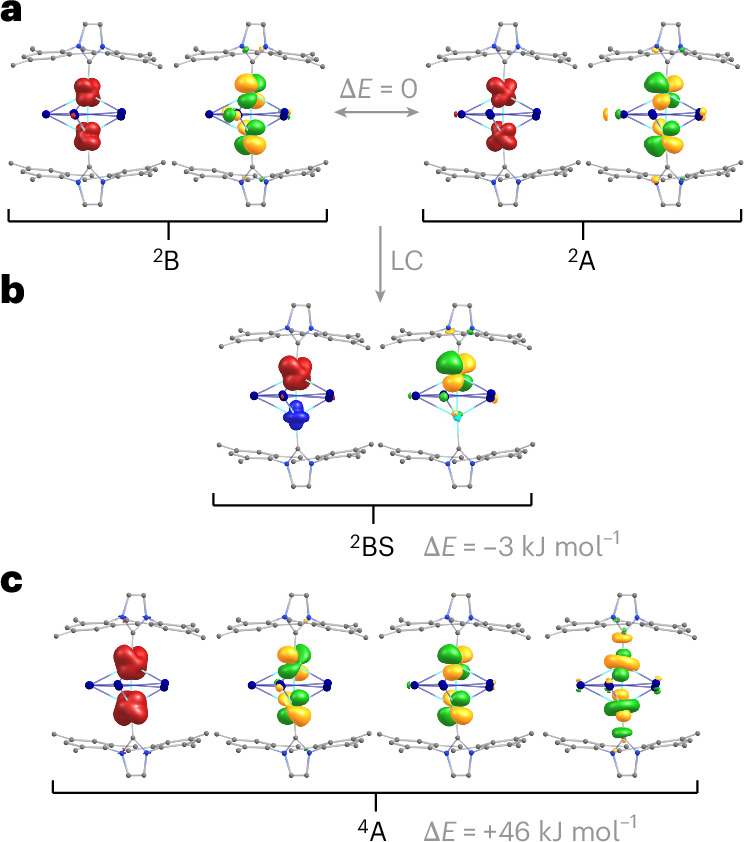
Spin densities and natural orbitals with occupations close to 1.0, for four possible group states of [{IMesCo}_2_Bi_5_] at level PBE/def2-TZVP. **a**, Spin densities (red/blue) and natural orbitals (green/yellow) for the two doublet ground states ^2^B and ^2^A (*S* = 1/2). **b**, Spin density (red/blue) and natural orbital (green/yellow) for the broken symmetry doublet state ^2^BS (*S* = 1/2), resulting from a linear combination (LC) of the ^2^B and ^2^A states. **c**, Spin density (red/blue) and natural orbitals (green/yellow) for the quartet ground state ^4^A (*S* = 3/2). Contours are drawn at 0.004 a.u. for the spin densities and at 0.04 a.u. for the natural orbitals. Energy differences (grey) are given with respect to the ^2^B state.

Neutral species with electronic doublet states (*S* = 1/2) are obtained by removal of one electron either from MO 126a, ^2^A, or from MO 123b, ^2^B (shown in Fig. 4c); further, a broken-symmetry state with the unpaired electron residing mainly at one Co atom can be constructed by linear combination of both. These three states are energetically almost degenerate (Table 1). For each state, the spin density as well as the corresponding natural orbitals with occupation eigenvalues close to 1.0 (that is, occupation by 1 electron) are shown in Fig. 5. The high similarity of these natural orbitals (Fig. 5) with the canonical orbitals (Fig. 4) indicates high stability of the electronic structure upon removal of one electron. As a consequence, the HOMO–LUMO (lowest unoccupied molecular orbital) gaps for the doublet states are small (~0.2 eV). Also, the changes in the structure parameters are rather small (Table 1), as the electron is removed from orbitals that are non-bonding. The energetically lowest quartet state (*S* = 3/2), ^4^A, is obtained by occupying 123b, 126a and 124b with one electron each. This state can be ruled out as the ground state, first due to its energy, which is 46 kJ mol^−1^ above the doublets and second due to its structural parameters. The Co–Co distance is more than 0.10 Å too long, and the C–Co–Co angle is ~10° smaller than in the experimentally determined structure. We thus conclude that in [{IMesCo}_2_Bi_5_] (1) the HOMO and HOMO-1, and so the two Co(*d*) orbitals in plane with the IMes rings, are occupied together with three electrons (instead of four for the anion).

Notably, results obtained with hybrid functionals, such as PBE0 instead of PBE, are much less conclusive (Supplementary Fig. 11 and Supplementary Table 4).

Finally, the title compound [{IMesCo}_2_Bi_5_] is compared with related, reported structures [{IMesNi}_2_P_5_] (ref. ^68^), [{(C_5_H_5_)Mo}_2_As_5_] (ref. ^11^) and [{(1,2,4-*t*Bu_3_C_5_H_2_)Mo}_2_Sb_5_] (ref. ^12^). As above, we first consider the corresponding anions, which are electronic closed-shell systems for all four compounds, and then identify the orbital that is only singly occupied for the neutral species. [{IMesNi}_2_P_5_]^−^ has two valence electrons more than [{IMesCo}_2_Bi_5_]^−^; consequently, the LUMO of [{IMesCo}_2_Bi_5_]^−^ becomes the HOMO of [{IMesNi}_2_P_5_] (see upper part of Supplementary Fig. 12). In [{(C_5_H_5_)Mo}_2_As_5_]^−^, which has the same valence electron count as [{IMesCo}_2_Bi_5_]^−^, the order of the MO energy levels is notably different: degenerate HOMO and HOMO-1 are closely related to HOMO-5 and HOMO-6 of [{IMesCo}_2_Bi_5_]^−^ (the same is true for [{(1,2,4-*t*Bu_3_C_5_H_2_)Mo}_2_Sb_5_]^−^); in turn, the MOs of [{(C_5_H_5_)Mo}_2_As_5_]^‒^ that are equivalent to HOMO/HOMO-1 of [{IMesCo}_2_Bi_5_]^−^ (HOMO-15/HOMO-16 and HOMO-12/HOMO-13) are notably stabilized by bonding interactions with the (C_5_H_5_)^−^ ligands, which is a clear difference from the title compound. For each neutral compound, the natural orbital with occupation eigenvalue close to 1.0 (that is, the one representing the unpaired electrons in the doublet state) is shown in the lower part of Supplementary Fig. 12, for the X-ray structure data. For [{IMesNi}_2_P_5_], the shape is virtually identical to that of the corresponding HOMO of the (structure-optimized) anionic species, *C*_2_ symmetry will be kept for the neutral species (apart from crystal packing effects). For [{IMesCo}_2_Bi_5_], its shape is very close to that of the *C*_1_ broken-symmetry state (Fig. 5). For [{(C_5_H_5_)Mo}_2_As_5_] (and [{(1,2,4-*t*Bu_3_C_5_H_2_)Mo}_2_Sb_5_]), the shape is similar to one column of the degenerate HOMO of the *D*_5*h*_-symmetric anion, the symmetry of which is obviously reduced to at least *C*_2*v*_ for the neutral species. Overall, these compounds of type [{LM}_2_Pn_5_] may be described as an (aromatic) Pn_5_^−^ ring with substantial orbital interactions to the embedding {LM}_2_^+^ entity, where the latter exhibits one unpaired electron distributed over the two M atoms. A more detailed, extended comparison is provided in Supplementary Fig. 13.

## Conclusion

To summarize, we have presented the synthesis and characterization of [{(IMes)Co}_2_(µ,η^5^:η^5^-Bi_5_)]. This compound displays the long sought-for, planar five-membered Bi_5_^−^ ring, and heaviest 6π-aromatic all-metal (C_5_H_5_)^−^ analogue, thereby completing the series of Pn_5_^−^ ligand-containing compounds that have been known for the lighter elements of group 15 for several years. As confirmed by sophisticated quantum chemical calculations and µ-SQUID analysis, 1 is a mixed-valence, open-shell Co^0^/Co^I^ complex, perfectly suited for coordination chemistry with the Bi_5_^−^ ligand. The compound is overall neutral—an exceptional finding in the context of synthetic chemistry with Zintl anions and an excellent precondition for follow-up chemistry. Its isolation was achieved by using a solvent that is atypical in this context, *o*-DFB.

The synthesis of polypnictogen compounds have long been driven by readily available starting materials, in particular white phosphorus, and now bismuth is catching up as deeper understanding into the chemistry of polybismuthides becomes more apparent. The isolation of the Bi_5_^−^ ring in a metal complex in the solid state, and also its further observation in solution, is an invaluable next step. The (C_5_H_5_)^−^ ligand is embedded into the framework of inorganic chemistry, and now this example comprising the all-bismuth analogue opens up several avenues of study that were previously not possible with molecular bismuth compounds. We envisage that the isolation of 1 can help develop the chemistry of Pn_5_^−^ ligands as the unique physical and electronic properties of bismuth, a safe-to-handle heavy metal, can be introduced and combined with transition metal and organometallic complexes in an unprecedented way.

## Methods

### General methods

All reactions were carried out under a dry, argon atmosphere using standard Schlenk line or glovebox techniques. Solvents were purified as appropriate: en (Roth, >99.5 %) and *o*-DFB (Apollo Scientific, 99%) were refluxed over CaH_2_ for 24 h, distilled and stored on 4 Å molecular sieves, tetrahydrofuran or tetrahydrofuran-d8 (Aldrich, 99.5%;) was refluxed over potassium for 24 h, distilled and stored on 4 Å molecular sieves, and *n*-hexane (Sigma Aldrich, >95%) was refluxed over Na for 24 h, distilled and stored on 4 Å molecular sieves. BiI_3_ was prepared by heating a stoichiometric mixture of Bi (ChemPur, 99.5%) and I_2_ (Thermo Scientific 99.5%) under an argon atmosphere to 180 °C for 5 h. [CoIMes_2_Cl], [K(crypt-222)]_2_Bi_2_ and [K(crypt-222)]_2_(TlBi_3_)∙0.5en were prepared as described previously; all analytical data accord with the reported ones^28,37,76^.

### Reaction of [K(crypt-222)]_2_Bi_2_ and BiI_3_

[K(crypt-222)]_2_Bi_2_ (62 mg, 0.05 mmol) was dissolved in en (5 ml) and treated with BiI_3_ (15 mg, 0.025 mmol) as a solid. The resultant dark-green suspension was stirred for 3 h and then filtered. The filtrate solution was analysed by ESI-MS (Fig. 1a).

### Synthesis of [{IMesCo}_2_Bi_5_] (1)

[K(crypt-222)]_2_(TlBi_3_)∙0.5en (30 mg, 0.02 mmol) and [(IMes)_2_CoCl] (15 mg g, 0.04 mmol) were combined in a brown glass sample vial and dissolved in *o*-DFB (3 ml) in a glovebox. After 3 h a brown suspension had formed. The suspension was filtered through a syringe filter and subsequently layered with *n*-hexane (3 ml). After storage at −20 °C for 1 month, small dark-brown plank-shaped crystals formed at the bottom of the Schlenk tube (due to the low quantities obtained it was not possible to accurately weigh the material obtained owing to the error in the balance, we can only estimate for a mass of approximately 5 mg, 15% yield).

### Single-crystal X-ray diffraction data

The datasets were collected on a Bruker D8 Quest with microfocus source emitting MoKαradiation (λ = 0.71073 Å) and a Photon 100 detector at *T* = 100 K. The structures were solved by dual space methods of SHELXT-2018/2 within the Olex2-1.3 software and refined using least-squares procedures on a *F*^2^ with SHELXL-2018/3 in Olex2^77^. General crystallographic data are listed in Supplementary Table 2; supplementary structural images are shown in Supplementary Figs. 7 and 8.

### μ-XRF

All μ-XRF studies were performed with a Bruker M4 Tornado, equipped with an Rh-target X-ray tube and a silicon drift detector. The emitted fluorescence photons were detected with an acquisition time of 180 s. Quantification of the elements was achieved through deconvolution of the spectra. Results are shown in Fig. 2d and Supplementary Table 3.

### ESI-MS

All mass spectra were recorded with a Thermo Fisher Scientific Finnigan linear ion trap Fourier-transform (LTQ-FT) mass spectrometer in negative ion mode, ESI(−). The solutions were injected into the spectrometer with gastight 250 µl Hamilton syringes by syringe pump infusion. All capillaries within the system were washed with dry *o*-DFB 2 h before and at least 10 min in between parameters were used: spray voltage 3.6 kV, capillary temperature 290 °C, capillary voltage −42 V, tube lens voltage −137 V, sheath gas 38, sweep gas 0, auxiliary gas 8. Overview spectra are shown in Supplementary Figs. 2 and 3; assignable high-resolution mass peaks are shown in Supplementary Figs. 4 and 5.

### µ-SQUID measurements

A single crystal of compound 1 was placed in the vicinity of an array of µ-SQUIDs on a chip and thermalized using Apiezon grease. The sample was cooled down to 30 mK base temperature in a dilution refrigerator equipped with a three-dimensional vector magnet. The magnetic field could be applied in any direction of the µ-SQUID plane with a precision better than 0.1° by separately driving three orthogonal coils. The low-temperature *M*(*H*) measurements (0.03–4.5 K) were performed on the single crystals at different field sweep rates between 0.002 and 0.128 T s^−1^ with a time resolution of approximately 1 ms. An easy axis, although not prominently found in this sample, was estimated by using a transverse field method^78,79^. Angle-dependent *M*(*H*) curves at 30 mK were also obtained to map the derivatives d*M*(*H*)/d*H* with the direction of applied field (Supplementary Fig. 9), but the anisotropy is not clearly visualized due to the nearly paramagnetic nature and possibly isotropic *J* = *S* = 1/2.

### Quantum chemical calculations

Calculations were performed with TURBOMOLE^48^^,^^80^. The PBE functional^49^ was used with dhf-TZVP basis sets^50^ together with corresponding effective core potentials^51^ for Bi, neglecting and self-consistently including the effect of SOC^53^. Note that the results obtained for the anion in 1 with the functional PBE0 are much less conclusive: while structure parameters for the anion are quite similar to those obtained with PBE, the doublet states suffer from elongated Co–Co distances of +12, +13 and +49 pm for ^2^A, ^2^B and ^2^BS, respectively, and +60 pm for the quartet state. More details are given in Supplementary Table 4. We further note that also our complete active space self-consistent field calculations yield similarly unsatisfactory results, at least for the technically treatable active spaces. The global minimum structure of Bi_5_^−^ was determined with a genetic algorithm procedure^52^. For bond analysis, LMOs were generated by a Pipek–Mezey MO localization procedure^56^, and the number of unpaired electrons was calculated with a Mulliken population analysis^75^. Magnetically induced current densities were obtained with the GIMIC code^57,81^ using the perturbed density from TURBOMOLE^82^. The *g* factors were calculated within the all-electron scalar relativistic variant of the exact two-component decoupling approach^83^ using corresponding basis sets of triple-zeta valence quality^84^. Complete active space self-consistent field calculations were done with ORCA, version 5.0.4^85,86^.

## Online content

Any methods, additional references, Nature Portfolio reporting summaries, source data, extended data, supplementary information, acknowledgements, peer review information; details of author contributions and competing interests; and statements of data and code availability are available at 10.1038/s41557-024-01713-8.

## Supplementary information

Supplementary Information Supplementary information, figures and tables for quantum chemical studies, ESI-MS, light microscopy, single-crystal diffraction and refinement, micro-X-ray fluorescence spectroscopy and magnetic studies: Supplementary Figs. 1–13 and Tables 1–4.

Supplementary Data 1 Crystallographic data for 1 (CCDC-2362176).

Supplementary Data 2 Cartesian coordinates and total energies of all computed structures.

Supplementary Data 3 Original magnetic data of compound 1 and origin file of Fig. 3.

## Data Availability

The structure of compound 1 was determined by single-crystal X-ray diffraction. Crystallographic data for the structure reported in this Article have been deposited at the Cambridge Crystallographic Data Centre, under deposition number CCDC-2362176 (1). A copy of the data can be obtained free of charge via https://www.ccdc.cam.ac.uk/structures/. The Cartesian coordinates of all optimized structures and the respective SCF energies are provided as a supplementary data file. The files comprise all necessary data for reproducing the values. All non-default parameters for the computational studies are given in the Supplementary Information together with the corresponding references of the used methods. For the default parameters of TURBOMOLE, such as the convergence criteria for structure optimizations, please see the manual at https://www.turbomole.org. The data collected in the µ-SQUID measurement underlying Fig. 3 and the respective discussion are provided in a supplementary data file.

## Code availability

The TURBOMOLE quantum program suite is available from https://www.turbomole.org, see also refs. ^48^^,80^, and the GIMIC code (version 2.1.4 commit 3a5f0eb2019) is available via GitHub at https://github.com/qmcurrents/gimic, open source; see also refs. ^57^^,81^. The GitHub repository also includes a sample input for GIMIC. The ORCA quantum chemical program package is available at https://orcaforum.kofo.mpg.de/app.php/dlext/.
