# Supplementary material for: Isolation of a planar π-aromatic Bi5− ring in a cobalt-based inverse-sandwich-type complex
Source: Nat Chem. 2025 Jan 20;17(4):547–55. doi: 10.1038/s41557-024-01713-8 (PMC11964920; doi:10.1038/s41557-024-01713-8)
Supplement: Supplementary file 1 — Supplementary information, figures and tables for quantum chemical studies, ESI-MS, light microscopy, single-crystal diffraction and refinement, micro-X-ray fluorescence spectroscopy and magnetic studies: Supplementary Figs. 1–13 and Tables 1–4. [file 41557_2024_1713_MOESM1_ESM.pdf]

nature chemistry

Supplementary information https://doi.org/10.1038/s41557-024-01713-8

Isolation of a planar π-aromatic Bi_5^- ring in a cobalt-based inverse-sandwich-type complex

In the format provided by the authors and unedited

Table of Contents

1 Quantum Chemical Calculations of Bi_5^- 2

2 Electrospray ionization mass spectrometry (ESI-MS) investigations of the reaction solutions during the formation of [{IMesCo}$_2$Bi$_5$] (1) 4

2.1 Methods 4

2.2 Mass spectra of the reaction solution 4

3 Light microscopic Images of the Single crystals 8

4 Single-Crystal Diffraction and Refinement Data of [{IMesCo}$_2$Bi$_5$] (1) 9

4.1 Crystal Measurement and Refinement Details 9

5 Supplementary Structural Figures 10

6 Micro-X-Ray Fluorescence Spectroscopy (µ-XFS) 12

7 Magnetic studies: Derivative-field angle map from µ–SQUID $M(H)$ loops 13

8 Quantum Chemical Calculations of [{IMesCo}$_2$Bi$_5$] (1) and Related Compounds 14

9 References 21

1 Quantum Chemical Calculations of Bi_5^-

Quantum chemical calculations were done with TURBOMOLE (details of the computations are provided in the Methods section of the main document).[1] An unbiased search of the global minimum of Bi_5^- was carried out with a genetic algorithm procedure.[2] In **Supplementary Table 1**, the Bi–Bi distance is listed for the most favourable structure, the planar ring (D_{5h}), as well as its energetic preference over the second stable isomer, a capped butterfly (C_{2v}), see **Figure 1b** in the main document, for several methods.

Supplementary Table 1 | Bi–Bi distance in the D_{5h} structure of Bi_5^- and energetic preference over the C_{2v} structure. The dhf-TZVP basis[3] together with Dirac-Hartree-Fock effective core potentials[4] was used unless explicitly mentioned otherwise.

**Method**	**d(Bi-Bi)/pm**	**ΔE/kJmol^{-1}**
PBE[5]	287.7	30
BP86[6,7]	287.9	39
TPSS[8]	286.6	21
PBE0[9]	283.5	20
B3LYP[10]	287.7	48
TPSSh[11]	285.0	18
HF[12]	282.9	19
MP2[13]	281.0	22
PBE, 2c-ECP,[14] dhf-TZVP-2c basis[3]	291.3	66
PBE, scalar X2C,[15] x2c-TZVPall-2c basis[16]	286.7	32
PBE, 2c-X2C,[17] x2c-TZVPall-2c basis[16]	290.3	70

The electronic structure of the Bi_5^- ring is analogous to that of $(C_5H_5)^-$. A selection of the valence MOs of both species are shown in **Figure 1d** in the main document. Full valence MO diagrams of both species are given in **Supplementary Figure 1**.

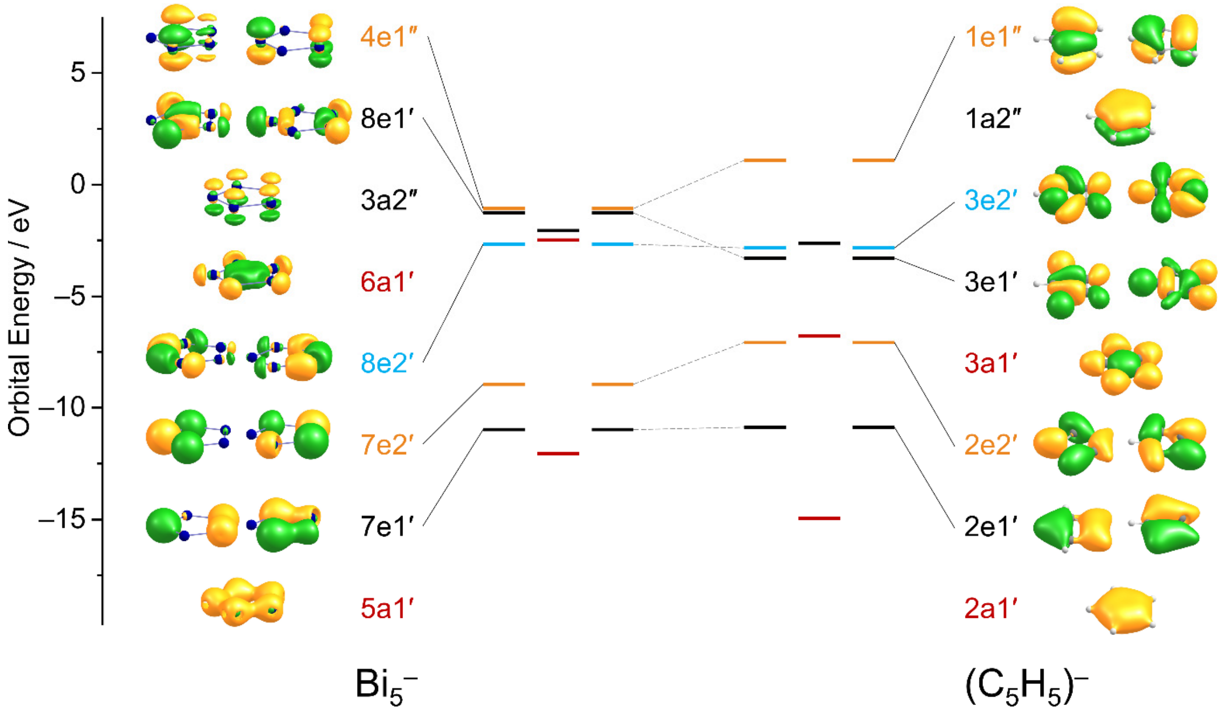

Supplementary Figure 1 | Energies and amplitudes of valence MOs of Bi_5^- (left) and $(C_5H_5)^-$ (right) obtained with the PBE functional and dhf-TZVP basis sets. Different superatomic MO types (albeit of a 2D molecule) are specified by different colors of bars and Mulliken symbols: s-type (red), p-type (black), d-type (orange), f-type (blue); contours are drawn at ±0.04 a.u.

Localized molecular orbitals were calculated for Bi_5^- and the model compound $[Bi_5(CoC_3N_2H_4)_2]$. Bi_5^- has 16 6p electrons that occupy the eight HOMOs and are energetically well separated from the 6s orbitals by ca. 7 eV. On the other hand, it has five symmetry-equivalent Bi–Bi contacts. It is obvious that a simple localized description is problematic. In fact, the Pipek-Mezey localization procedure[18] for these MOs yields five LMOs corresponding to Bi–Bi σ-bonds, while the π-system is distributed over the remaining three LMOs, of which only two are equivalent, see **Figure 1c** in the main document. The intrinsic delocalization is in line with the comparably high ring current observed for this system as well as for its lighter homologues and related $(C_5H_5)^-$ which all show the same features concerning localization.

2 Electrospray ionization mass spectrometry (ESI-MS) investigations of the reaction solutions during the formation of [{IMesCo}$_2$Bi$_5$] (1)

2.1 Methods

All mass spectra were recorded with a Thermo Fischer Scientific Finnigan LTQ-FT spectrometer in negative ion mode, ESI(–). The solutions were injected into the spectrometer with gastight 250 µL Hamilton syringes by syringe pump infusion. All capillaries within the system were washed with dry *o*-DFB 2 hours before and at least 10 min in between parameters were used: Spray Voltage: 3.6 kV, Capillary Temp: 290 °C, Capillary Voltage: −42 V, Tube lens Voltage: −137 V, Sheath Gas: 38, Sweep Gas: 0, Auxiliary Gas: 8. Overview spectra after 5 minutes and 2h reaction time are shown in **Supplementary Figure 2** and **Supplementary Figure 3**, respectively. Assignable high-resolution mass peaks are shown in **Supplementary Figure 4** and **Supplementary Figure 5**.

2.2 Mass spectra of the reaction solution

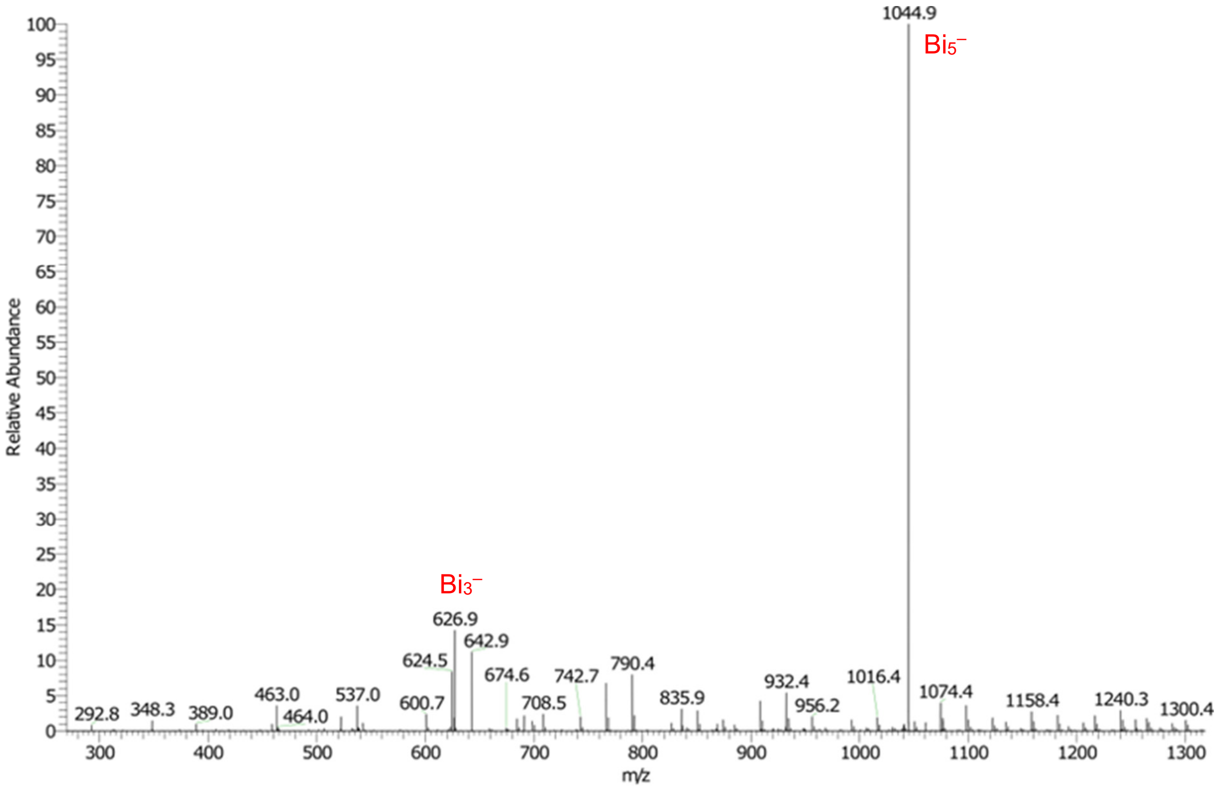

Supplementary Figure 2 | Overview ESI(−) mass spectrum recorded after 5 min reaction time. Identifiable mass peaks are indicated, which demonstrate the rapid and selective formation of Bi_5^- in the reaction mixture.

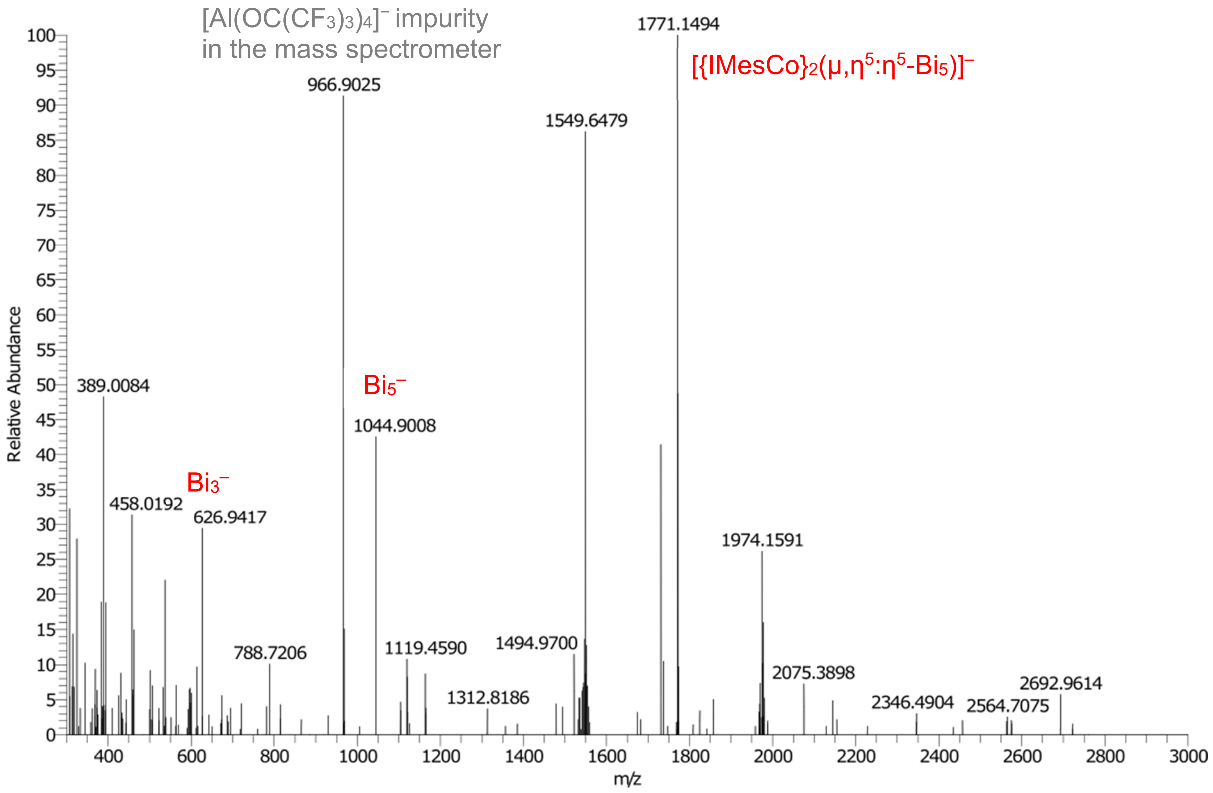

Supplementary Figure 3 | Overview ESI(−) mass spectrum recorded after 2 h reaction time. Most dominant identifiable mass peaks are indicated, with the predominant one demonstrating the abundance of complex **1** (in the form of its anion).

.

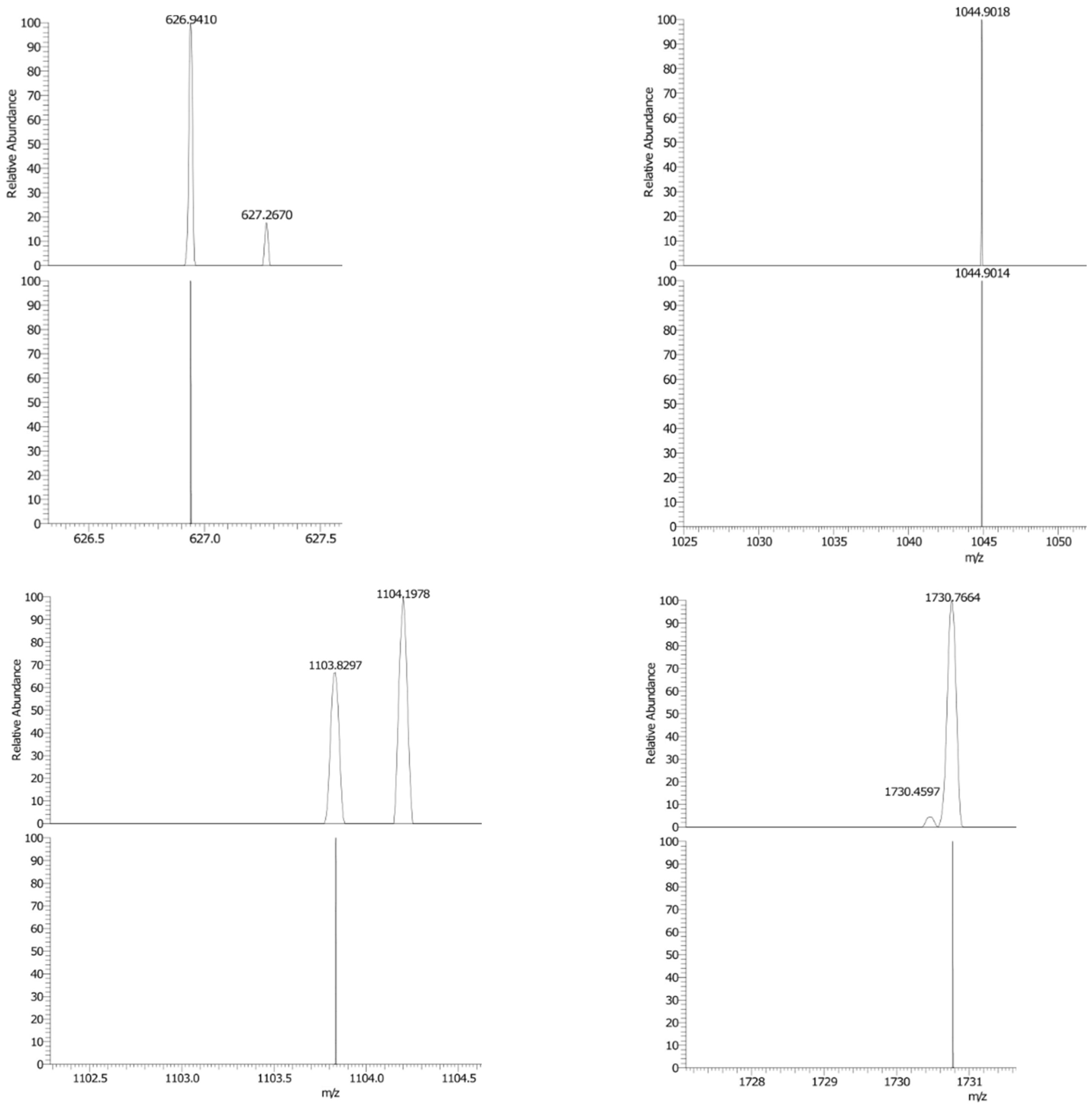

Supplementary Figure 4 | High-resolution ESI(–) mass spectra recorded after 2 h reaction time showing homometallic or heterometallic anions. The high-resolution spectra (upper curve: measured, below: simulated) indicate the existence of polybismuthide anions Bi_3^- (top left) and Bi_5^- (top right), as well as heterometallic fragments $(Bi_5Co)^-$ (bottom left) and $(Bi_8Co)^-$ (bottom right) of complex **1**.

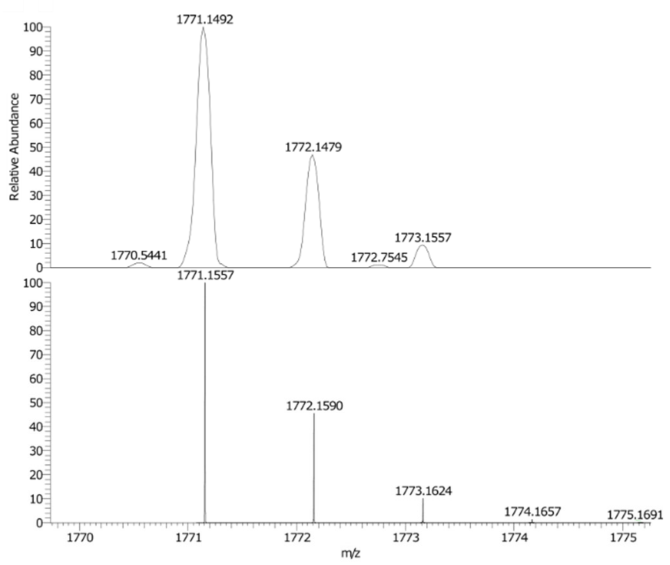

Supplementary Figure 5 | High-resolution ESI(–) mass spectrum in negative ion mode recorded after 2 h reaction time showing the anion of 1. The high-resolution spectrum refers to the existence of **1** in solution, detected as anion under ESI-MS conditions, prior to crystallization. Topmost: measured, below: simulated.

3 Light microscopic Images of the Single crystals

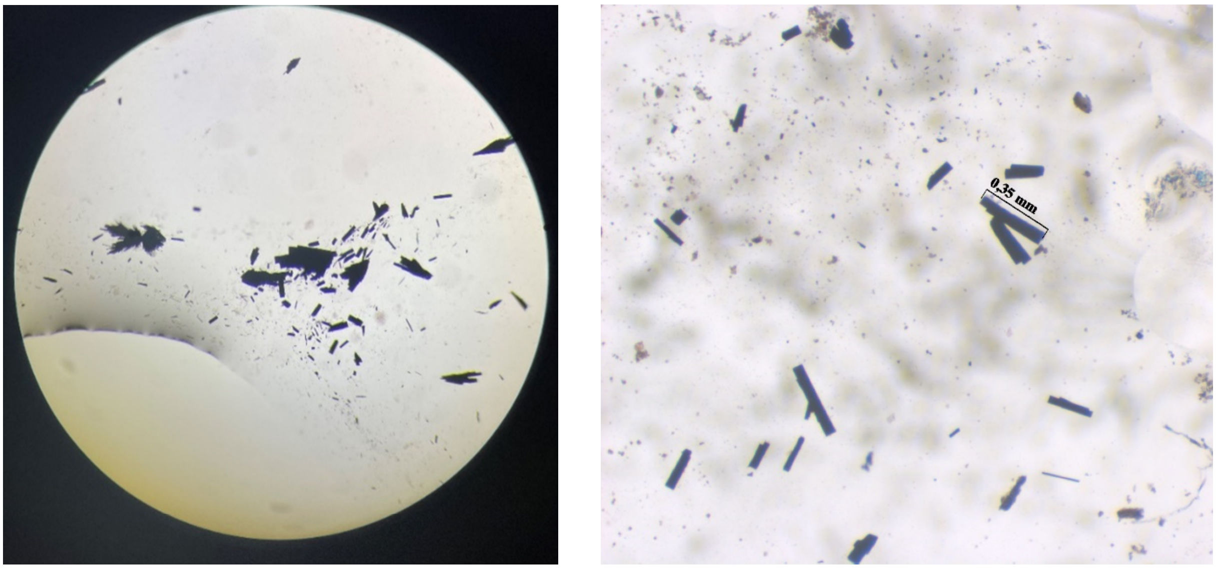

Supplementary Figure 6 | Crystal photographs of compound 1 taken through a light microscope. The scale bar in the figure on the right-hand side indicates the typical size of the single crystals.

4 Single-Crystal Diffraction and Refinement Data of [{IMesCo}$_2$Bi$_5$] (1)

4.1 Crystal Measurement and Refinement Details

The data sets were collected on a Bruker D8 Quest with microfocus source emitting MoK_α radiation (λ = 0.71073 Å) and a Photon 100 detector at T = 100 K. The structures were solved by dual space methods of SHELXT-2018/2 within the Olex2-1.3 software and refined using least-squares procedures on a F^2 with SHELXL-2018/3 in Olex2.[19] General crystallographic data are listed in **Supplementary Table 2**.

Supplementary Table 2 | Crystal data and details of the structure determination of compound 1.

**Compound**	**1**
Empirical formula	C_{42} H_{48} Bi_5 Co_2 N_4
Chemical formula	[{IMesCo}$_2$(μ,η^5:η^5-Bi$_5$)]
Emp. formula weight [g mol^{-1}]	1771.60
Temperature [K]	100
Crystal color, shape	metallic dark black, block
Crystal system	orthorhombic
Space group (no)	*Pccn* (56)
a [Å]	17.1783(12)
b [Å]	15.058(3)
c [Å]	17.048(4)
V [Å^3]	4409.7(14)
Z	4
ρ_{calc} [g cm^{-3}]	2.668
μ [mm^{-1}]	20.646
F(000)	3188
Crystal size	0.35 × 0.05 × 0.10
Radiation	Mo Kα
2θ range [°]	4.318-66.258
Index ranges	$-26 \le h \le 26$, $-23 \le k \le 23$, $-25 \le l \le 0$
Absorption correction type	Multi-scan
Reflections collected	29599
Ind. reflections / R(int) / R(sigma)	8349 / 0.0392 / 0.0351
Restraints / parameters	0 / 246
Final R indexes [$I \ge 2\sigma(I)$]	R_1 = 0.0269, wR_2 = 0.0552
Final R indexes [all data]	R_1 = 0.0362, wR_2 = 0.0574
Goodness-of-fit on F^2	1.066
Max peak / hole [e Å^{-3}]	1.470 / –1.793
CCDC number	2362176

5 Supplementary Structural Figures

All non-hydrogen atoms were refined using anisotropic displacement parameters. All hydrogen atoms were refined by using a riding model. Absorption correcting was carried out using MULTISCAN. Supplementary structural figures are shown in **Supplementary Figure 7** and **Supplementary Figure 8**. The structures were drawn with DIAMOND.[20] They are shown with displacement ellipsoids at the 50% probability level for non-hydrogen atoms.

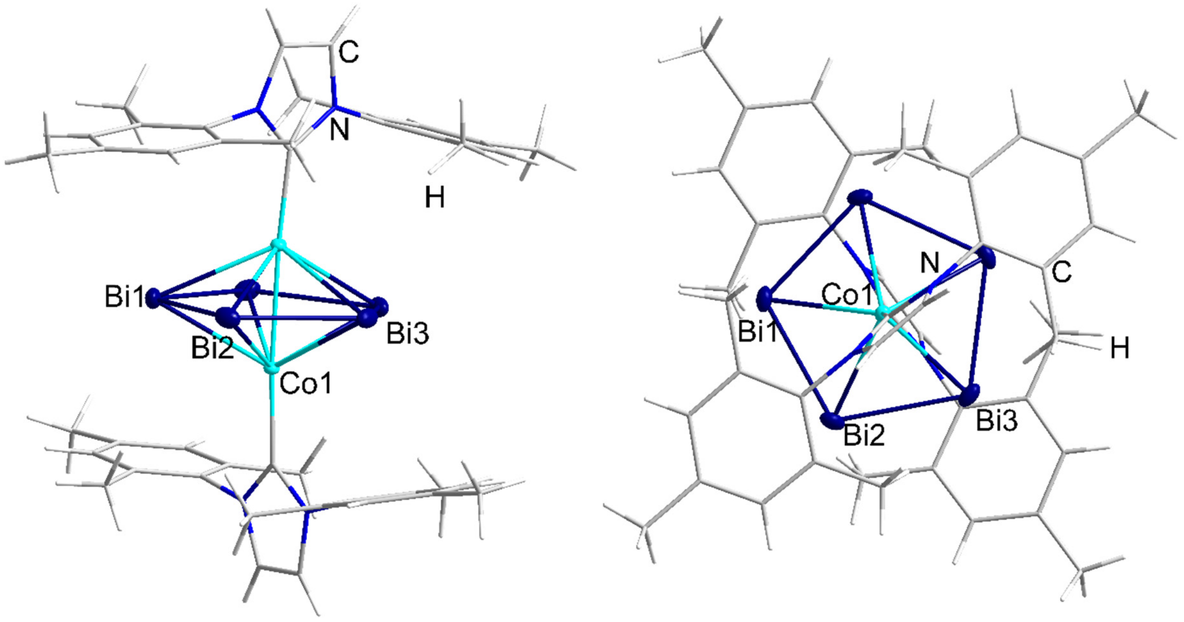

Supplementary Figure 7 | Two views of the molecular structure of compound 1 with all H atoms shown and atoms of the asymmetric unit labelled. Displacement ellipsoids are shown with 50% probability for non-H atoms; organic groups are given in wire mode. Color code: Bi (dark blue), Co (turquoise), C (grey), N (blue), H (white).

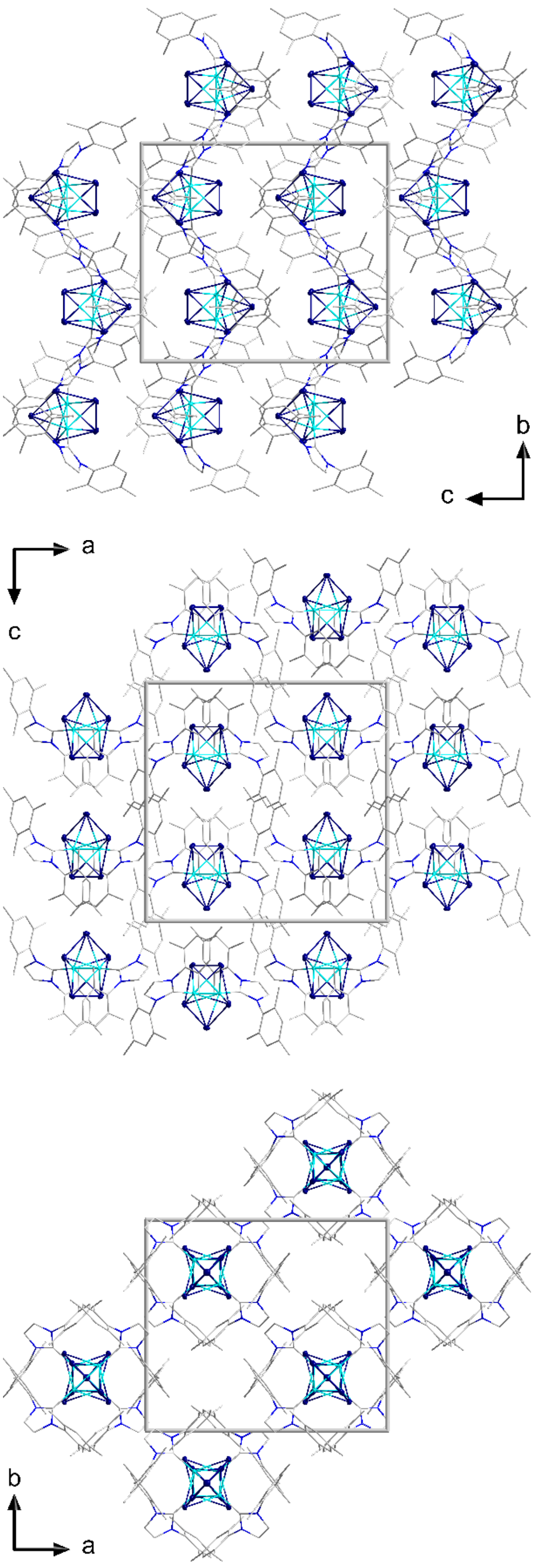

Supplementary Figure 8 | Views of the extended unit cell of compound 1 viewed along crystallographic axes *a*, *b*, and *c* (from top). Displacement ellipsoids are shown with 50% probability for non-H atoms; H atoms are not shown for clarity. Color code: Bi (dark blue), Co (turquoise), C (grey), N (blue).

6 Micro-X-Ray Fluorescence Spectroscopy (μ-XFS)

All μ-XFS measurements were performed on a Bruker M4 Tornado, equipped with an Rh-target X-ray tube and a silicon drift detector. Quantification of the elements is achieved through deconvolution of the spectra. The results are summarized in **Supplementary Table 3**. The rhodium content from the target is omitted from the quantification results. The spectrum for single crystals of compound **1** is shown in **Figure 2d** in the main document along with the results of the deconvolution algorithm.

Supplementary Table 3 | Summary of the micro-X-ray fluorescence spectroscopy results.

Element	Atomic Nr.	Mass[%]	Atom Cont. Obs. [%]	Atom cont. Calc. [%]
**Bi**	83	89.9	71.6	71.4
**Co**	27	10.1	28.4	28.6

7 Magnetic studies: Derivative-field angle map from μ–SQUID *M*(*H*) loops

Employing the 3D vector magnet, *M*(*H*) loops at T = 30 mK were measured for different directions of applied field within the μ–SQUID plane. The derivatives d*M*/d*H* of the positive half-cycle are plotted with different in-plane angles as a d*M*/d*H* (H_x,H_y) color map, as shown in **Supplementary Figure 9**. Due to least magnetic coupling to the μ-SQUIDs, a certain direction (close to the *y* axis in this case) shows a white patch, i.e., the least magnitude of the derivative. Such a map is usually helpful to indicate the anisotropy in a system with large spin *J* (or *S*) and coupling (for example a dimer of 4f ions). However, here the anisotropy in the system is not clearly realized probably because of the isotropic $J = S = 1/2$ in this compound.

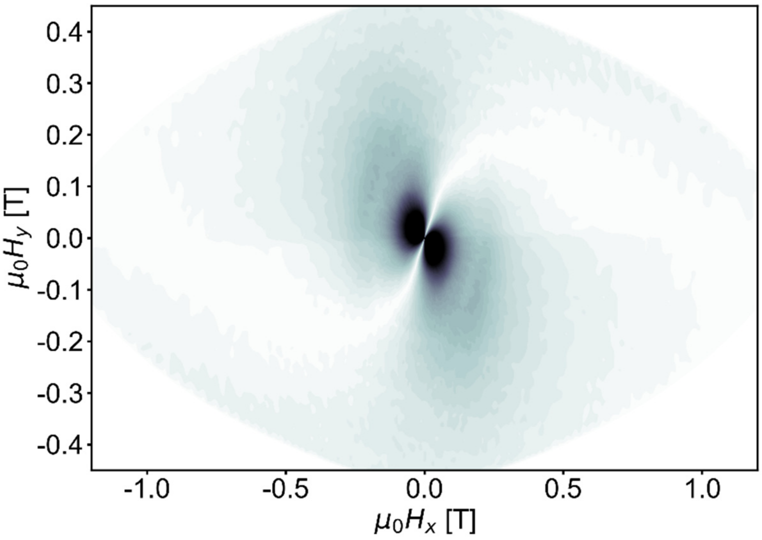

Supplementary Figure 9 | d*M*/d*H* (H_x,H_y) map obtained from positive half of the *M*(*H*) loops measured by a μ-SQUID at different in-plane angles.

8 Quantum Chemical Calculations of [{IMesCo}$_2$Bi$_5$] (1) and Related Compounds

For the calculation of localized molecular orbitals, we considered the model compound [Bi$_5$(CoC$_3$N$_2$H$_4$)$_2$]. Here, the highest 23 MOs are well separated from the lower ones by 3 eV. Resulting LMOs sorted by increasing energy expectation values are shown in **Supplementary Figure 10**. LMOs 7–11 represent the five σ-bonds in the Bi$_5$ ring, LMOs 12–14 are the π-system, now slightly delocalized towards the Co atoms. The overall character of the Bi_5^- ring thus is still preserved. We further observe one LMO per Co-C σ-bond (1,2), one per Co-C π-bond (16,17), one for the Co-Co bond (15), and six LMOs (18–23) being located almost exclusively at one of the Co atoms (three per atom). LMOs 3-6 represent the π-systems of the two C$_3$N$_2$ rings.

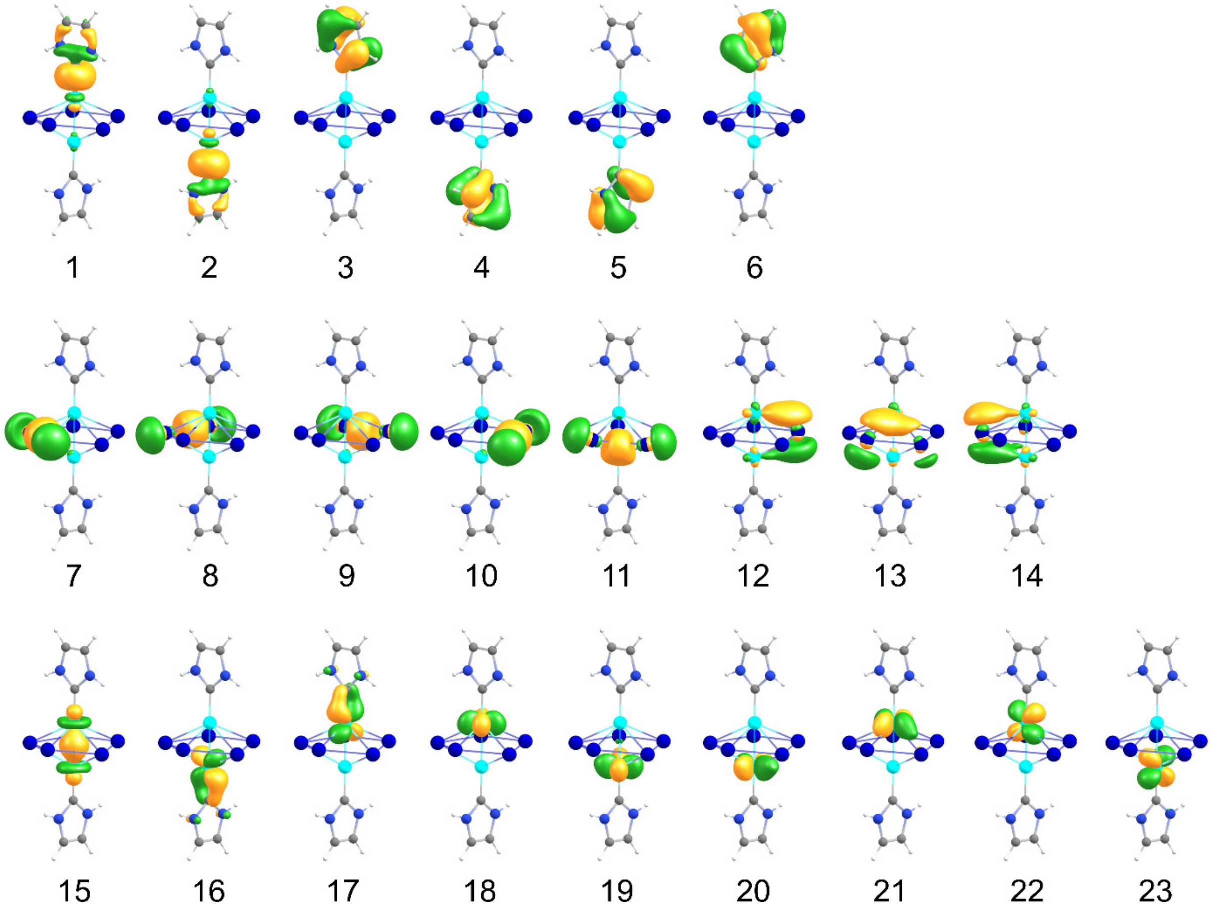

Supplementary Figure 10 | Localized molecular orbitals (LMOs) of [Bi$_5$(CoC$_3$N$_2$H$_4$)$_2$] resulting from a Pipek-Mezey procedure applied to the energetically highest 23 canonical MOs. LMOs are listed in the sequence of their energy expectation values, beginning with the lowest one (#1) at the top left corner and ending with the highest one to the bottom right (#23).

The title compound [{IMesCo}$_2$Bi$_5$] (**1**) was additionally calculated with the hybrid functional PBE0. Images of MOs of the corresponding anion are shown in **Supplementary Figure 11** together with that obtained with PBE, structure parameters of the optimized structures of the anion and four electronic states of the neutral species as well as data concerning the electronic structure are listed in **Supplementary Table 4**. Most striking differences in the MOs is the high admixture of Bi contributions to the five highest occupied MOs in case of PBE0 and the shape of the HOMO. With PBE0 it is the binding MO between the Co atoms, which is HOMO-5 with PBE, whereas the two HOMOs with PBE are non-binding combinations of Co(d) orbitals.

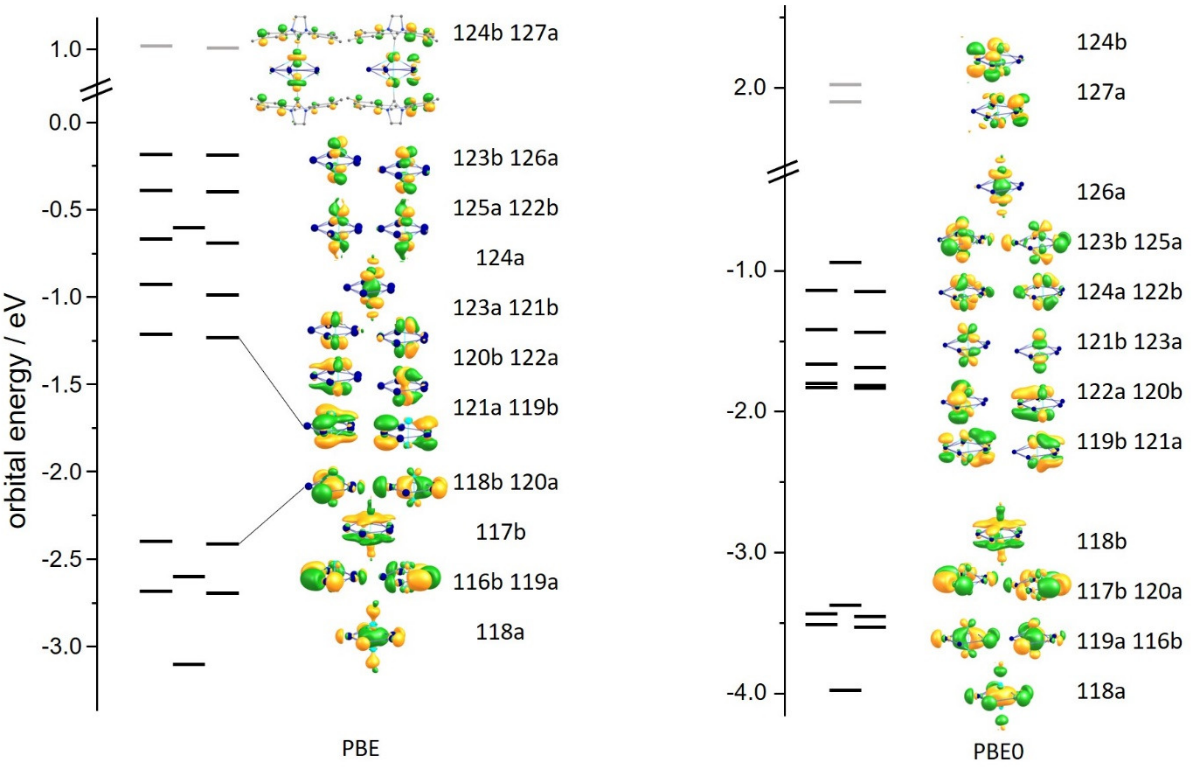

Supplementary Figure 11 | Valence MOs of [{IMesCo}$_2$Bi$_5$]$^-$ obtained with PBE and PBE0 functionals. The MOs calculated with PBE are shown to the left, those with PBE0 are shown to the right. Contours are drawn at 0.04 a.u.

Supplementary Table 4 | Geometric and electronic structure data of [{IMesCo}$_2$Bi$_5$] (1) and the hypothetical anion [{IMesCo}$_2$Bi$_5$]$^-$ obtained with the functional PBE0. Column labelling: Co–Co, Co–Bi and Bi–Bi are the (ranges of) distances between the corresponding atoms, C–Co–Co labels the bend of the Co–Co axis and the Co–C axes at the two Co atoms. *E* is the energy relative to the ^{2}A state in kJ mol^{-1}, in the following column, the S^2 values and their deviations from the value for the pure doublet/quartet are given, Gap denotes the HOMO-LUMO Gap and N_{ue} the number of unpaired electrons according to a Mulliken analysis[21] at the Co atoms and at the {Bi$_5$} ring. Row labelling: M$^-$ denotes the anionic (diamagnetic) species, the subsequent lines denote neutral species, in detail: In ^{2}A, the occupation of orbital 126a (HOMO within the irrep a) is reduced from 2 to 1, in ^{2}B that of 123b (HOMO within irrep b), ^{2}BS is a broken symmetry-state with overall one unpaired electron. In ^{4}A the unpaired electrons reside in 126a, 123b and 124b.

	Co–Co [Å]	C–Co–Co [°]	Co–Bi [Å]	Bi–Bi [Å]	*E* [kJ mol^{-1}]	S^2 (S^2–S[S+1])	Gap [eV]	N_{ue} (Co/Co/Bi5)
X-ray	2.52	176	2.75 – 2.79	2.90 – 2.91	–	–	–	–
Anion	2.48	177	2.68 – 2.78	2.87 – 2.88		0		0.0/0.0/0.0
^{2}A	2.60	170	2.67 – 2.90	2.89 – 2.92	0	1.423 (0.673)	2.691	0.77/0.77/–0.46
^{2}B	2.61	178	2.68 – 3.05	2.91 – 2.93	–30	1.541 (0.791)	1.803	1.08/1.08/–1.01
^{2}BS	2.97	179/169	2.78 – 3.19	2.92 – 2.95	–141	2.562 (1.812)	1.711	2.08/–1.94/0.86
^{4}A	3.12	170	2.79 – 3.25	2.92 – 2.93	–143	4.563 (0.813)	2.030	2.02/2.02/–0.98

Whereas for the anion PBE0 structure parameters agree reasonably well with PBE and also with the experimental data for the neutral species, unphysically large deviations in the Co–Co distance and in the Co–Bi distances are observed for the neutral species, i.e., as soon as one of the HOMOs of the anion is depleted. This is particularly true for the energetically seemingly favourable states ^{2}BS and ^{4}A, for which the Co–Co distance is calculated 45 pm and 60 pm too long. This comes along with high spin contaminations. Apparently PBE0 and (probably) all other hybrid functionals as well as calculations basing on Hartree-Fock wave functions fail in the description of this compound.

Additionally, CASSCF calculations were performed on the simplified system $Bi_5(CoC_3N_2H_4)_2$ with more economical computational settings. The ECP-10[22] for Co and ECP-78[23] for Bi by the Stuttgart group were employed. For Co, the ECP-10-MDF basis set[22] was used, while for the other atoms def-SV(P) bases[24] were chosen. First, it was made sure that the chosen computational settings for the model system led to similar results as the original settings for the original system. This means that with PBE the C_2 symmetry of the system in the electronic and

molecular structure is maintained also without symmetry constraints (calculated Mulliken populations indeed are the same for both atoms). It was further made sure that just like in the original system the electronic structure obtained with PBE0 breaks the symmetry by showing different Mulliken populations on the Co atoms and that a geometry optimization with PBE0 heavily distorts the symmetry of the structure.

For CASSCF calculations (ORCA V. 5.0.4[25,26]), an informed guess regarding the active space and the starting orbitals is necessary for a meaningful result. This is non-trivial in this case, as there are several orbitals on both Co atoms of s and d character and several p orbitals on the Bi ring, which all might be of relevance for the Co–Co distance. Additionally, we encountered convergence problems both for the CI- and the macro-iterations, especially for larger active spaces. The largest one we tried contained 23 electrons in 15 orbitals. In the following, we discuss a case where we managed to obtain convergence.

Investigation of the Löwdin orbital compositions of the unrestricted natural orbitals of a PBE calculation of the model system made it plausible to place orbitals in the active space which are dominated by d(Co) orbitals and p_z(Bi) orbitals (parallel to the Co–Co bond). It was decided to put 7 electrons and 7 orbitals in the active space as a starting point, in order to keep computational effort moderate and to obtain a converged wave function. One doublet state without state-averaging was calculated.

With the starting orbitals provided by ORCA as a default, this CASSCF(7,7) calculation did converge, maintaining a very similar Mulliken population on both Co atoms. Here, the active space consists out of orbitals dominated by d(Co) and p_z(Bi) orbitals, as expected. Despite this seemingly plausible wave function, the subsequent geometry optimization resulted in a symmetry-broken electronic structure (different Mulliken populations for the two Co atoms), a distorted molecular structure, and a Co–Co distance of ca. 362 pm, which is obviously far too long. Next, the CASSCF(7,7) wave function in the PBE structure was used as a starting point for a calculation with 9 electrons in 8 active orbitals. Again, a plausible wave function was obtained, but the geometry optimization once more resulted in two different Mulliken populations on Co, a distorted molecular structure, and a Co–Co distance of ca. 355 pm. Therefore, our CASSCF calculations tend towards a symmetry breaking of the wave function, which in turn leads to a distorted molecular structure and far too large Co–Co distances, just like HF admixtures in the DFT calculations do. As we did not succeed in obtaining a stable CASSCF wave function and molecular geometry which qualitatively fit to the experimental findings, no further insights were gained from the CASSCF calculations.

Supplementary Figure 12 shows the MOs of structures related to [{IMesCo}$_2$Bi$_5$] (**1**), namely [{IMesNi}$_2$P$_5$][27] and [{(C$_5$H$_5$)Mo}$_2$As$_5$][28] (similar situation as for [{(1,2,4-*t*Bu$_3$C$_5$H$_2$)Mo}$_2$Sb$_5$]$^-$),[29] in relation to those of the former, in all cases for the anionic closed-shell structures. Albeit the mixing between orbitals of the five-membered ring and the transition metal differs for the three compounds, the identification of related MOs is still possible and indicated with connecting lines in **Supplementary Figure 13**. The Ni compound has two more valence electrons than the Co compound, thus the LUMO of the latter becomes the HOMO of the former, LU(Co)→HO(Ni). The Mo compound has six valence electrons less than the Co compound, which leads to the following consequences. The analogues of HO(Co) and HO–1(Co), which are located exclusively at the transition metal atoms, are no longer occupied. HO–2(Co) and HO–3(Co), which are dominated by d(Co) with (small) admixtures from the neighbouring C atoms of the IMes groups significantly change their character in the Mo compound: HO–12(Mo) and HO–13(Mo) also show contributions from the d orbitals and from the ligand, but here dominated by the latter ((C$_5$H$_5$)$^-$). Further, HO–5(Co) and HO–6(Co) become HO(Mo) and HO–1(Mo), while HO–4(Co) becomes HO–2(Mo).

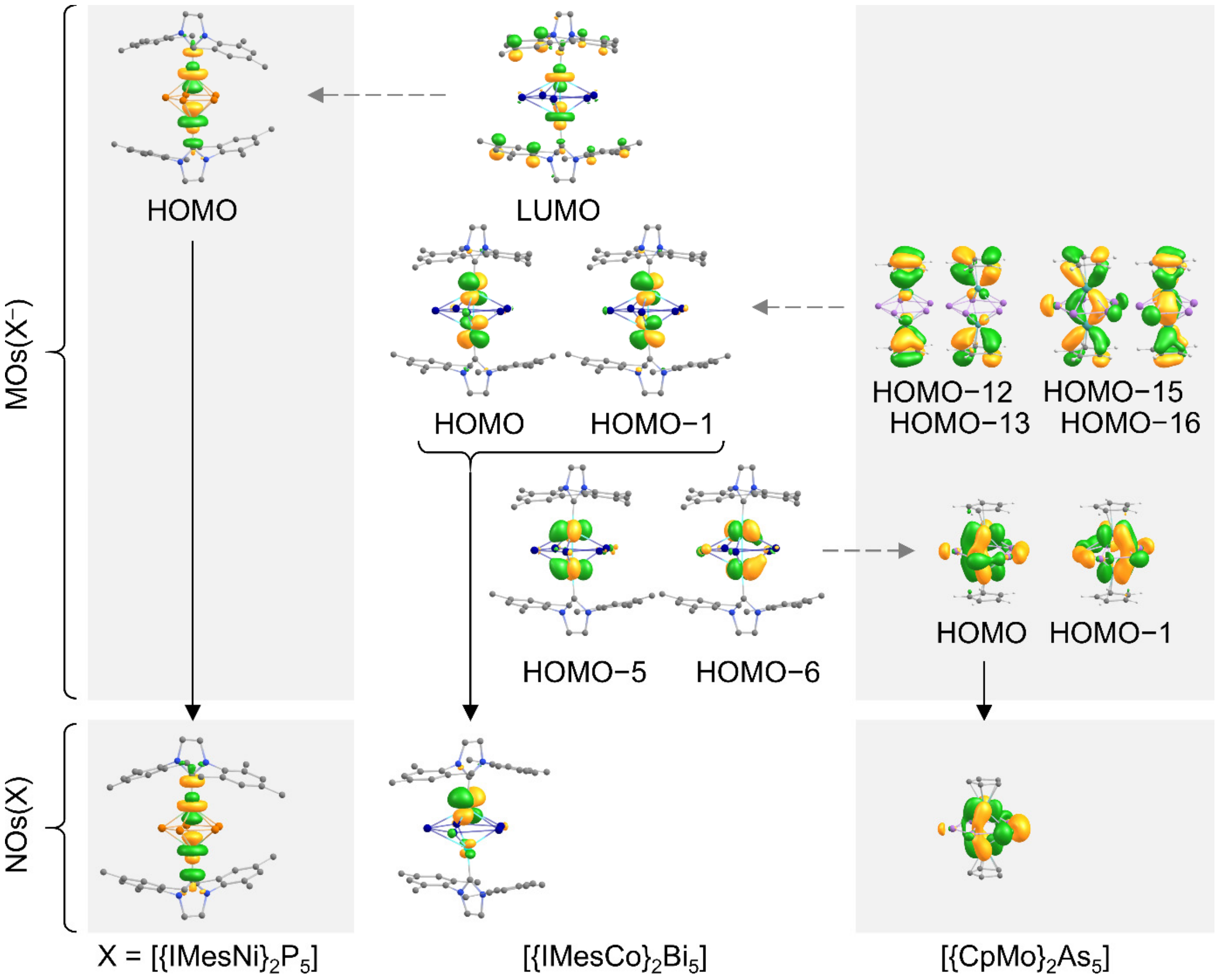

Supplementary Figure 12 | Comparison of frontier orbitals and natural orbitals of calculated species [{IMesNi}$_2$P$_5$]$^-$, [{IMesCo}$_2$Bi$_5$]$^-$, and [{(C$_5$H$_5$)Mo}$_2$As$_5$]$^-$. 'X' denotes the neutral species, 'X$^-$' denotes the corresponding anions. Upper part: Frontier molecular orbitals (MOs) of [{IMesCo}$_2$Bi$_5$]$^-$, [{IMesNi}$_2$P$_5$]$^-$ and [{(C$_5$H$_5$)Mo}$_2$As$_5$]$^-$ in their optimized structures. Lower part: Natural orbitals (NOs) with occupation eigenvalue close to one for their neutral counter parts (doublet states) in the X-ray structure.

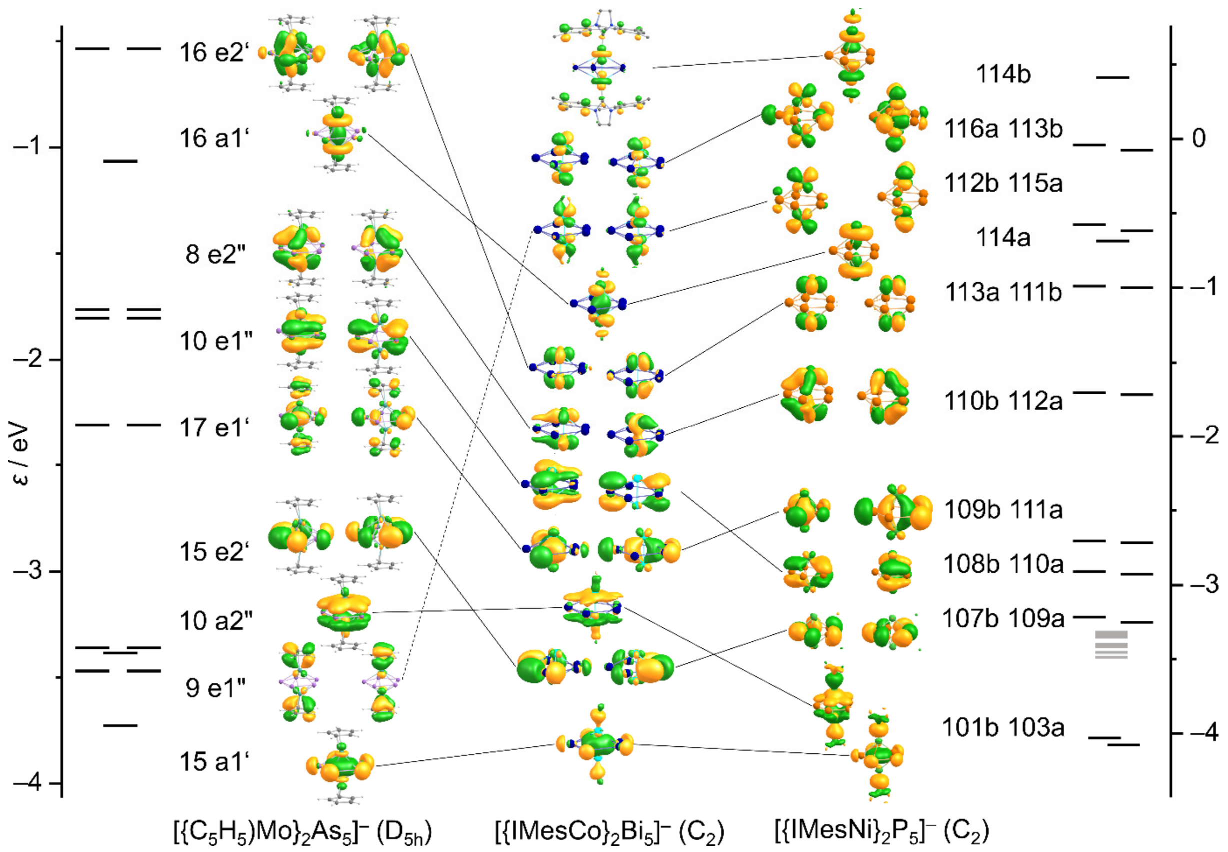

Supplementary Figure 13 | Images of occupied valence orbitals of structures related to hypothetic anion [{IMesCo}$_2$Bi$_5$]$^-$. The results for [{IMesCo}$_2$Bi$_5$]$^-$ are shown in the middle, between those of related compounds, namely hypothetical anion [{(C$_5$H$_5$)Mo}$_2$As$_5$]$^-$ (left; similar situation as for [{(1,2,4-*t*Bu$_3$C$_5$H$_2$)Mo}$_2$Sb$_5$]$^-$, which is not shown here) and hypothetical anion [{IMesNi}$_2$P$_5$]$^-$ (right); for [{IMesCo}$_2$Bi$_5$]$^-$ additionally the LUMO is shown (middle, top), for [{IMesNi}$_2$P$_5$]$^-$ a bundle of MOs between –3 and –4 eV with contributions solely from the IMEs groups is listed only in the energy level diagram (grey bars). IMes groups are omitted for clarity, except for the LUMO of the Co compound, which is the only orbitals shows significant contributions from this groups.

9 References

1. TURBOMOLE pre-version 7.7 2022 (University of Karlsruhe and Forschungszentrum Karlsruhe, 1989–2007, and TURBOMOLE, since 2007, accessed 13 February 2022); http://www.turbomole.com.
2. Sierka, M. *et al.* Unexpected structures of aluminum oxide clusters in the gas phase. *Angew. Chem. Int. Ed.* **46**, 3372–3375 (2007).
3. Weigend, F. & Baldes, A. Segmented contracted basis sets for one- and two-component Dirac-Fock effective core potentials. *J. Chem. Phys.* **133**, 174102 (2010).
4. Metz, B., Stoll, H. & Dolg, M. Small-core multiconfiguration-Dirac-Hartree-Fock-adjusted pseudopotentials for post-d main group elements: Application to PbH and PbO. *J. Chem. Phys.* **113**, 2563–2569 (2000).
5. Perdew, J. P., Burke, K. & Ernzerhof, M. Generalized Gradient Approximation Made Simple. *Phys. Rev. Lett.* **77**, 3865–3868 (1996).
6. Perdew, J. P. Density-functional approximation for the correlation energy of the inhomogeneous electron gas. *Phys. Rev. B: Condens. Matter Mater. Phys.* **33**, 8822–8824 (1986).
7. Becke, A. D. Density-functional exchange-energy approximation with correct asymptotic behaviour. *Phys. Rev. A: At., Mol., Opt. Phys.* **38**, 3098–3100 (1988).
8. Tao, J., Perdew, J. P., Staroverov, V. N. & Scuseria, G. E. Climbing the density functional ladder: Nonempirical meta–generalized gradient approximation designed for molecules and solids. *Phys. Rev. Lett.* **91**, 146401 (2003).
9. Perdew, J. P. & Ernzerhof, M. Rationale for mixing exact exchange with density functional approximations. *J. Chem. Phys.* **105**, 9982–9985 (1996).
10. Lee, C., Yang, W. & Parr, R. G. Development of the Colic-Salvetti correlation-energy formula into a functional of the electron density. *Phys. Rev. B: Condens. Matter Mater. Phys.* **37**, 785–789 (1988).
11. Staroverov, V. N., Scuseria, G. E., Tao, J. & Perdew, J. P. Comparative assessment of a new nonempirical density functional: Molecules and hydrogen-bonded complexes. *J. Chem. Phys.* **119**, 12129–12137 (2003).
12. Häser, M. & Ahlrichs, R. Improvements on the direct SCF method. *J. Comput. Chem.* **10**, 104–111 (1989).
13. Weigend, F. & Häser, M. RI-MP2: First derivatives and global consistency. *Theor. Chem. Acc.* **97**, 331–340 (1997).
14. Baldes, A. & Weigend, F. Efficient two-component self-consistent field procedures and

gradients: Implementation in Turbomole and application to Au-20. *Mol. Phys.* **111**, 2617–2624 (2013).

15. Peng, D., Middendorf, N., Weigend, F. & Reiher, M. An efficient implementation of two-component relativistic exact-decoupling methods for large molecules. *J. Chem. Phys.* **138**, 184105 (2013).
16. Pollak, P. & Weigend, F. Segmented Contracted Error-Consistent Basis Sets of Double- and Triple-ζ Valence Quality for One- and Two-Component Relativistic All-Electron Calculations. *J. Chem. Theory Comput.* **13**, 3696–3705 (2017).
17. Franzke, Y. J., Middendorf, N. & Weigend, F. Efficient implementation of one- and two-component analytical energy gradients in exact two-component theory. *J. Chem. Phys.* **148**, 104110 (2018).
18. Mezey, P. G. & Pipek, J. A fast intrinsic localization procedure applicable for ab initio and semiempirical linear combination of atomic orbital wave functions. *J. Chem. Phys.* **90**, 4916–4926 (1989).
19. Sheldrick, G. M. SHELXT-integrated space-group and crystal-structure determination. *Acta Crystallogr. A Struct. Chem.* **71**, 3–8 (2015).
20. K. Brandenburg, Diamond, Crystal Impact GbR, Bonn, Germany, 2021
21. Mulliken, R.S. Electronic Population Analysis on LCAO-MO Molecular Wave Functions. *J. Chem. Phys* **23**, 1833–1840 (1955).
22. Dolg, M.; Wedig, U.; Stoll, H.; Preuss, H. Energy-adjusted *ab initio* pseudopotentials for the first row transition elements, *J. Chem. Phys*. **86**, 866-872 (1987).
23. Kuechle, W.; Dolg, M.; Stoll, H.; Preuss, H. *Ab initio* pseudopotentials for Hg through Rn, *Mol. Phys*. **74**, 1245-1263 (1991).
24. Schäfer, A.; Horn, H.; Ahlrichs, R. Fully optimized contracted Gaussian basis sets for atoms Li to Kr. *J. Chem. Phys.* **97**, 2571-2577 (1992).
25. Neese, F. Software update: The ORCA program system—Version 5.0 *Wiley Interdiscip. Rev.: Comput. Mol. Sci.* 12, e1606 (2022).
26. F. Neese, ORCA – An *ab initio*, DFT and semiempirical SCF-MO package, V. 5.0.4, MPI für Kohleforschung, Mülheim an der Ruhr, Germany, 2023.
27. Hierlmeier, G., Coburger, P., van Leest, N. P., de Bruin, B. & Wolf, R. Aggregation and Degradation of White Phosphorus Mediated by N-Heterocyclic Carbene Nickel(0) Complexes. *Angew. Chem. Int. Ed.* **59**, 14148–14153 (2020).
28. Rheingold, A. L., Foley, M. J. & Sullivan, P. J. "Triple-Decker Sandwich" with a Planar As_5 Ring. Synthesis and Crystal Structure of CpMo[μ-(η^4-As_5)]MoCp. *J. Am Chem. Soc.*

104, 4727–4729 (1982).

29. Breunig, H. J., Burford, N. & Rösler, R. Stabilization of a pentastibacyclopentadienyl ligand in the triple-decker sandwich complexes. *Angew. Chem. Int. Ed.* **39**, 4148–4150 (2000).
